# Plant-Based ZnO Nanoparticles for Green Nanobiocontrol of a Highly Virulent Bacterial Leaf Blight Pathogen: Mechanistic Insights and Biocompatibility Evaluation

**DOI:** 10.3390/nano15131011

**Published:** 2025-06-30

**Authors:** Preeda Chanthapong, Duangkamol Maensiri, Paweena Rangsrisak, Thanee Jaiyan, Kanchit Rahaeng, Atcha Oraintara, Kunthaya Ratchaphonsaenwong, Jirawat Sanitchon, Piyada Theerakulpisut, Wuttipong Mahakham

**Affiliations:** 1 Department of Biology, Faculty of Science, Khon Kaen University, Khon Kaen 40002, Thailand; jirapat@kku.ac.th (P.C.); rpaweena@kkumail.com (P.R.); thaneeja@kkumail.com (T.J.); kanchit.ra@kkumail.com (K.R.); piythe@kku.ac.th (P.T.); 2 School of Biology, Institute of Science, Suranaree University of Technology, Nakhon Ratchasima 30000, Thailand; 3 Department of Microbiology, Faculty of Science, Khon Kaen University, Khon Kaen 40002, Thailand; atcha@kku.ac.th; 4 Research and Academic Services Division, Faculty of Science, Khon Kaen University, Khon Kaen 40002, Thailand; phikra@kku.ac.th; 5 Department of Agronomy, Faculty of Agriculture, Khon Kaen University, Khon Kaen 40002, Thailand; jirawat@kku.ac.th

**Keywords:** green synthesis, ZnO nanoparticles, *Centella asiatica*, antibacterial mechanism, ROS, biocompatibility, rice pathogen, sustainable disease management

## Abstract

Bacterial leaf blight (BLB), caused by *Xanthomonas oryzae* pv. *oryzae* (*Xoo*), poses a serious threat to rice cultivation. This study presents the green synthesis of zinc oxide nanoparticles (ZnO NPs) using an aqueous leaf extract of the medicinal plant *Centella asiatica* (L.) Urban and evaluates their potential as dual-function nanopesticides. The synthesized CA-ZnO NPs exhibited high crystallinity, a hexagonal to quasi-spherical morphology, and nanoscale dimensions (~22.5 nm), as confirmed by UV–Vis spectroscopy, XRD, FTIR, SEM, TEM, and SAED analyses. These nanoparticles demonstrated potent antibacterial activity against a highly virulent, field-derived Thai *Xoo* strain, with a minimum inhibitory concentration (MIC) of 8 µg/mL. Mechanistic investigations revealed substantial membrane disruption, intracellular nanoparticle penetration, and elevated reactive oxygen species (ROS) generation in treated cells. Cytotoxicity testing using human dermal fibroblasts (HDFs) revealed excellent biocompatibility, with no statistically significant reduction in cell viability at concentrations up to 500 µg/mL. In contrast, viability markedly declined at 1000 µg/mL. These findings underscore the selective antibacterial efficacy and minimal mammalian cytotoxicity of CA-ZnO NPs. Overall, CA-ZnO NPs offer a promising green nanopesticide platform that integrates potent antibacterial activity with biocompatibility, supporting future applications in sustainable crop protection and biomedical nanotechnology.

## 1. Introduction

Rice (*Oryza sativa* L.) is a staple food crop that is vital to global food and nutritional security, with nearly 90% of its production and consumption concentrated in Asia [1]. Thus, sustainable rice production is crucial to meeting the demands of the world’s growing population. However, rice cultivation faces numerous biotic stresses, among which bacterial leaf blight (BLB), caused by *Xanthomonas oryzae* pv. *oryzae* (*Xoo*), is one of the most devastating diseases in Asia [2,3]. BLB infections can result in significant yield losses, typically ranging from 20% to 70%, depending on the rice variety, infection stage, and environmental conditions [1,3]. Consequently, *Xoo* is consistently listed among the top ten most economically significant plant pathogenic bacteria due to its widespread impact on rice productivity and pathogenic complexity [4].

BLB management has become increasingly challenging due to the rapid evolution and diversification of *Xoo* populations, driven by host–pathogen co-evolution and agricultural practices [5,6]. Genomic and pathotypic analyses have revealed multiple virulent lineages, including strains capable of overcoming widely deployed resistance genes [6,7,8]. Although breeding for BLB resistance remains a key strategy, its durability is often undermined by the emergence of new *Xoo* pathotypes and the remarkable plasticity of the *Xoo* genome [5,9,10]. Moreover, the introgression of resistance genes frequently compromises important agronomic traits, necessitating trade-offs between disease resistance and yield or quality [11], while strong selective pressure from breeding programs further accelerates the adaptation of virulent strains [5].

*Xoo* is a Gram-negative bacterium that enters rice plants through wounds or hydathodes, colonizes the xylem vessels, and initiates systemic infection through multiple coordinated mechanisms, including the secretion of cell wall-degrading enzymes, biofilm-forming extracellular polysaccharides (EPSs), and virulence proteins delivered via the type III and type II secretion systems [11,12]. Among these, transcription activator-like (TAL) effectors play a pivotal role by modulating host susceptibility genes such as *OsSWEET11* and *OsSWEET14* to promote infection [2]. Genomic plasticity further enhances *Xoo* adaptability, with mobile genetic elements such as transposons and integrons driving the rapid evolution of virulence and resistance traits [12]. This has led to the emergence of aggressive field strains that overcome major resistance genes like *Xa4* through effector variation or regulatory mutations [2,12].

Chemical control represents a major component of BLB management and has commonly involved the application of bactericides such as bismerthiazol, streptomycin-based antibiotics, and copper compounds [11,13]. However, the effectiveness of these measures is rapidly declining due to the emergence of resistant *Xoo* populations, environmental toxicity concerns, and the broader issue of antibiotic resistance [13,14,15]. Recent studies have shown that *Xoo* can evade chemical treatment through multiple mechanisms, including enzymatic degradation, membrane modification, and metabolic reprogramming [11]. In particular, resistance to Zhongshengmycin (ZSM) has been associated with suppression of the pyruvate cycle (P cycle), a central metabolic pathway regulating antibiotic susceptibility. This suppression can be functionally reversed by exogenous alanine, which restores P cycle activity and sensitizes resistant strains to ZSM [16]. In addition, pathogenomic analyses have identified over 28 resistance-related genes, including multidrug efflux systems such as *MexCD-OprJ*, further underscoring the need for novel, sustainable alternatives to chemical control [12].

In addition to chemical approaches, biological control strategies employing antagonistic microbes such as *Bacillus* spp. and *Pseudomonas* spp. have been explored as eco-friendly alternatives for BLB management [11,17]. Nevertheless, biological control often faces limitations, including inconsistent field performance, narrow host specificity, and vulnerability to pathogen variability, which restrict the broader adoption of these methods [17]. Collectively, these challenges emphasize the urgent need for innovative and sustainable approaches to strengthen BLB management.

Nanotechnology has emerged as a promising frontier in agriculture, offering innovative strategies for disease control, crop protection, and productivity enhancement [18,19,20]. Among various inorganic materials, zinc oxide (ZnO) has garnered considerable interest due to its unique physicochemical properties, including high chemical stability, broad-spectrum antimicrobial activity, and biocompatibility [21,22]. Classified as a “generally recognized as safe (GRAS)” substance by the U.S. Food and Drug Administration (FDA), ZnO supports the potential biocompatibility of ZnO-based nanomaterials [21,22,23]. Capitalizing on these intrinsic properties, zinc oxide nanoparticles (ZnO NPs) have been developed to offer enhanced antimicrobial efficacy, improved physicochemical stability, and greater surface reactivity compared to their bulk counterparts [21,24]. Several studies have demonstrated that ZnO NPs exhibit potent antibacterial activity while maintaining low toxicity to human cells, making them attractive candidates for agricultural and biomedical applications [21,25,26,27].

The application of ZnO NPs for plant disease control has been documented in several economically important pathosystems. In bacterial diseases, ZnO NPs have demonstrated efficacy against *Ralstonia solanacearum* (bacterial wilt of tomato) [28,29] and *Xanthomonas campestris* pv. *vesicatoria* (bacterial leaf spot of tomato) [29,30]. Likewise, in fungal pathogens, ZnO NPs proved effective against *Fusarium oxysporum* (vascular wilt of tomato) [31], *Phytophthora infestans* (late blight of potato) [32], *Cercospora canescens* (leaf spot of mung bean) [33], and *Colletotrichum* spp. causing anthracnose in chili and coffee plants [34,35].

In rice pathology, ZnO NPs have demonstrated potential against fungal pathogens [36] and, more recently, against the bacterial blight pathogen (*Xoo*) [37,38,39,40,41]. Recent evidence also indicates that ZnO NPs can target *Xoo* through oxidative stress induction and membrane damage, suggesting their suitability as next-generation bactericides for BLB management [38,39,40,41]. While some studies have reported intracellular ROS generation in *Xoo* cells following nanoparticle treatment [38,39], comprehensive mechanistic investigations focusing on highly virulent, field-derived strains remain scarce. Previous work has largely involved laboratory strains or isolates without detailed pathogenicity characterization, leaving critical gaps in understanding the interactions between ZnO NPs and aggressive *Xoo* populations under controlled conditions [42].

ZnO NPs can be synthesized via physical, chemical, or biological methods [43]. While physical and chemical synthesis routes yield particles with uniform size and high purity, they often involve toxic reagents, high energy input, and pose environmental hazards [43,44]. In contrast, green synthesis using plant extracts offers a sustainable, cost-effective, and eco-friendly alternative [44,45]. Bioactive compounds present in plant extracts, such as flavonoids, terpenoids, and saponins, can act as natural reducing and stabilizing agents during nanoparticle formation [23,24,44,45].

*Centella asiatica* (L.) Urb., commonly known as Gotu Kola, is rich in phytochemicals such as triterpenoids (e.g., asiaticoside, madecassoside), flavonoids, and phenolic compounds, which possess antioxidant, anti-inflammatory, and antimicrobial properties [46,47]. These bioactive constituents can act as capping and stabilizing agents and may influence the nucleation and growth of ZnO nanocrystals during green synthesis processes [48,49].

Therefore, this study aimed to synthesize ZnO NPs using *C. asiatica* leaf extract and evaluate their antibacterial activity against a highly virulent Thai field isolate of *Xoo*. Additionally, the study sought to elucidate the underlying antibacterial mechanism by directly visualizing intracellular ROS generation following nanoparticle treatment. This integrated approach, combining green synthesis with mechanistic insights, offers a promising strategy for sustainable bacterial leaf blight management in rice cultivation.

## 2. Materials and Methods

### 2.1. Preparation of Plant Extract and Phytochemical Screening

Fresh *C. asiatica* plants were obtained from the same central (Bang Lamphu) market in Muang District, Khon Kaen Province, Thailand, to ensure sourcing consistency. The leaves were thoroughly washed with tap water, followed by triple rinsing with double-distilled water. Approximately 20 g of fresh leaves were homogenized in 100 mL of deionized (DI) water and subsequently boiled at 80 °C for 20 min, resulting in an extract with an approximate concentration of 200 mg/mL. After cooling to room temperature, the mixture was filtered through Whatman No. 1 filter paper, and the resulting aqueous extract was stored at 4 °C for up to one week prior to use.

To ensure batch-to-batch reproducibility, the extraction protocol was standardized following the general principles of botanical standardization recommended by AOAC [50], including a fixed extraction ratio (1:5 *w*/*v*), temperature (80 °C), heating duration (20 min), and storage conditions (4 °C, ≤7 days). Qualitative phytochemical screening was performed following the method described in [51], and the antioxidant capacity of the extract was evaluated using the DPPH radical scavenging assay.

### 2.2. DPPH Radical Scavenging Assay

The antioxidant activity of the aqueous *C. asiatica* (CA) extract was assessed using a modified DPPH radical scavenging assay adapted from [52]. A 0.2 mM DPPH solution was prepared by dissolving 39.4 mg of DPPH powder (Sigma-Aldrich, St. Louis, MO, USA) in methanol (QRëC™, Auckland, New Zealand) and adjusting the final volume to 500 mL in a volumetric flask. In a 96-well microplate, 100 µL of sample solution (initial concentration 200 mg/mL) was serially diluted with methanol to obtain various concentrations. Subsequently, 100 µL of DPPH solution was added to each well. After incubation for 30 min at room temperature in the dark, absorbance was measured at 517 nm using a microplate reader (SpectraMax M5, Molecular Devices, San Jose, CA, USA). Methanol was used as the blank control, and ascorbic acid served as the positive control. The percentage of DPPH radical scavenging activity was calculated using the following equation:

DPPH radical scavenging activity (%) = ((A_control − A_sample)/A_control) × 100

where A_control and A_sample represent the absorbance values of the control and the sample, respectively. The IC_50_ value, defined as the sample concentration required to scavenge 50% of DPPH radicals, was determined by nonlinear regression analysis using GraphPad Prism version 8.0 (GraphPad Software, San Diego, CA, USA). All experiments were performed in triplicate, and the results were presented as mean ± standard deviation (SD).

### 2.3. Green Synthesis of CA-ZnO NPs Using Aqueous Leaf Extract of C. asiatica

**Figure 1:**
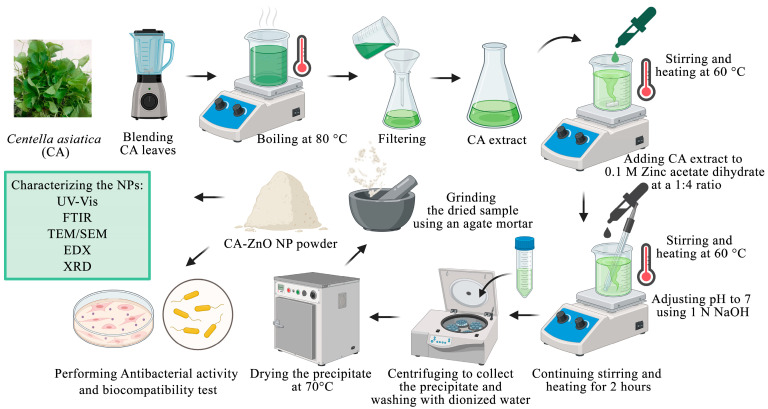
Schematic illustration of the green synthesis of CA-ZnO NPs using *C. asiatica* extract and their subsequent characterization and biological evaluation.

All chemicals used were of analytical grade. Zinc acetate dihydrate [Zn(O_2_CCH_3_)_2_(H_2_O)_2_] (Sigma-Aldrich, St. Louis, MO, USA) served as the zinc ion precursor. The precursor salt was dissolved in DI water prior to nanoparticle synthesis. The synthesis method was adapted from [53], with a modification of the precursor-to-plant extract ratio to 4:1 (*v*/*v*). Briefly, 80 mL of 0.1 M zinc acetate dihydrate solution was continuously stirred at room temperature, and 20 mL of aqueous plant extract was added dropwise. The pH was adjusted to 12 using 1 M NaOH solution (QRëC™, Auckland, New Zealand). The pH was adjusted to 12 using 1 M NaOH solution, based on previous studies reporting that pH 12 promotes optimal ZnO nanoparticle formation, yielding smaller particles with characteristic optical features and enhanced colloidal stability [54]. Nanoparticle formation was indicated by a visible change from a clear solution to a milky yellow suspension with a white precipitate. The resulting mixture was centrifuged at 6000 rpm (≈4500× *g*) for 15 min to collect the nanoparticle pellet, which was subsequently washed three times with deionized water, followed by one wash with ethanol, to remove residual ions and unreacted phytochemicals. The pale white pellets were oven-dried at 70 °C and ground into a fine powder using an agate mortar. The synthesized nanoparticles were designated as CA-ZnO NPs. The scheme illustrating the synthetic process of CA-ZnO NPs is shown in Figure 1.

### 2.4. Characterization of CA-ZnO NPs

#### 2.4.1. Optical Characterization by UV–Visible Spectroscopy

The synthesized CA-ZnO nanoparticles were dispersed in deionized water using an ultrasonic homogenizer to ensure uniform suspension. The nanoparticle suspension was then serially diluted with deionized water, and aliquots were transferred into quartz cuvettes for optical analysis. UV–visible absorption spectra were recorded over the range of 200–800 nm using a UV-Vis spectrophotometer (Spectrocord 200 Plus, Analytik Jena GmbH + Co. KG, Jena, Germany). For comparison, the optical properties of the aqueous *C. asiatica* extract were also measured under the same conditions.

#### 2.4.2. Morphological and Elemental Analysis by FE-SEM and TEM

The morphology and size distribution of the synthesized CA-ZnO NPs were analyzed using field emission scanning electron microscopy (FE-SEM) and transmission electron microscopy (TEM). For FE-SEM imaging, nanoparticles were dispersed in deionized water using an ultrasonic homogenizer, and a drop of the suspension was placed onto a carbon tape-coated SEM stub and air-dried. Imaging and elemental composition analysis were performed using a FEI Helios NanoLab G3 CX microscope (Thermo Fisher Scientific, Hillsboro, OR, USA) equipped with an energy dispersive X-ray (EDX) spectroscopy system.

For TEM analysis, a drop of the nanoparticle suspension was deposited onto a copper grid and allowed to dry at room temperature. TEM imaging and selected area electron diffraction (SAED) patterns were obtained using a FEI TECNAI G2 20 transmission electron microscope (Thermo Fisher Scientific, Hillsboro, OR, USA).

#### 2.4.3. Structural Characterization by X-Ray Diffraction (XRD)

The crystalline structure of the synthesized CA-ZnO NPs was analyzed using X-ray diffraction (XRD) with a Bruker D2 Phaser diffractometer (Karlsruhe, Germany). Powdered samples were placed onto a sample holder, and diffraction patterns were recorded using Cu Kα radiation (λ = 1.5406 Å) operated at 30 kV and 10 mA. Data were collected over a 2θ range of 20–80° at a scanning rate of 2°/min. The obtained diffraction peaks were compared with standard ZnO reference patterns (JCPDS Card No. 36-1451) for phase identification.

#### 2.4.4. Surface Functional Group Analysis by FTIR Spectroscopy

The surface functional groups of the phytochemicals present in the *C. asiatica* extract and the synthesized CA-ZnO nanoparticles were analyzed using Fourier-transform infrared (FTIR) spectroscopy (TENSOR27, Bruker Optics, Ettlingen, Germany) equipped with an attenuated total reflectance (ATR) accessory. Spectra were recorded in the range of 400–4000 cm^−1^ with a resolution of 4 cm^−1^.

#### 2.4.5. Dynamic Light Scattering (DLS) and Zeta Potential Analysis

The hydrodynamic size and surface charge (zeta potential) of CA-ZnO NPs were analyzed using a Zetasizer Nano ZS (Malvern Instruments, Malvern, UK). Nanoparticles were dispersed in deionized water, sonicated for 15 min, and transferred to disposable zeta cells for measurement at 25 °C. The results were reported as the Z-average size, polydispersity index (PDI), and zeta potential.

### 2.5. Antibacterial Activity, Mechanistic Investigations, and Cytocompatibility Assessment

#### 2.5.1. Bacterial Culture and Preparation

A virulent isolate of *Xoo* (NY1-1), previously isolated and characterized by [42], was used in this study. The bacterial cultures were maintained on peptone sucrose broth (PSB) and peptone sucrose agar (PSA) at 28 °C according to the method of [55].

The minimum inhibitory concentration (MIC) of CA-ZnO NPs against *Xoo* was determined using a broth microdilution method following the guidelines of the Clinical and Laboratory Standards Institute [56], with resazurin-based viability assessment according to the protocol described in [57]. CA-ZnO NPs were initially prepared at a concentration of 640 µg/mL in sterile deionized water and serially two-fold diluted in PSB medium to obtain final concentrations ranging from 1 to 512 µg/mL across a 96-well plate. *Xoo* cultures were prepared at an initial density of 1 × 10^8^ CFU/mL and diluted 1:150 in PSB medium to yield a final concentration of 1 × 10^5^ CFU/mL. A 100 µL aliquot of bacterial suspension was added to each well. Wells containing streptomycin sulfate (0.25–64 µg/mL) served as positive controls, and wells without nanoparticles served as growth controls. Plates were incubated at 28 °C for 48 h in the dark.

After incubation, 20 µL of 0.2% (*w*/*v*) resazurin solution (Sigma-Aldrich) was added to each well and incubated for an additional 2 h at 28 °C. Wells that retained a blue/purple color indicated no bacterial growth, whereas pink/colorless wells indicated bacterial viability. The MIC was defined as the lowest concentration of CA-ZnO NPs that prevented a color change. All assays were conducted in triplicate.

#### 2.5.2. Agar Disk Diffusion Assay

The antibacterial activity of CA-ZnO NPs was further evaluated using a modified Kirby–Bauer disk diffusion protocol based on [58]. PSA plates (~20 mL per 90 mm dish) were inoculated with mid-log phase *Xoo* cultures (1 × 10^8^ CFU/mL) using sterile swabs. Sterile filter paper disks were impregnated with CA-ZnO NPs at concentrations of 4, 8, 16, and 32 µg/disk and placed onto the inoculated plates. Sterile distilled water served as the negative control, while streptomycin (2 µg/disk) was used as the positive control. Plates were incubated at 28 ± 2 °C for 48 h. Zones of inhibition (ZOIs) were measured in millimeters using a vernier caliper. All experiments were performed in triplicate.

#### 2.5.3. Time–Kill Kinetics Assay

The time–kill kinetics assay was performed to evaluate the antibacterial activity of CA-ZnO NPs against *Xoo* over a 24 h period. A single fresh colony of *Xoo* was inoculated into PSB and incubated overnight at 28 °C with shaking at 180 rpm. The culture was then diluted in fresh PSB and grown until reaching the mid-logarithmic phase. A working inoculum of approximately 1 × 10^7^ CFU/mL was prepared and further adjusted with CA-ZnO NP suspensions to achieve a final bacterial concentration of approximately 1 × 10^6^ CFU/mL.

CA-ZnO NPs were applied at final concentrations corresponding to 0.25× MIC (2 µg/mL), 0.5× MIC (4 µg/mL), and 1× MIC (8 µg/mL). Each treatment group was incubated at 28 °C in sterile glass test tubes without shaking. At designated time points (0, 4, 8, 12, 16, 20, and 24 h), 1 mL aliquots were collected and bacterial growth was monitored by measuring optical density at 600 nm (OD_600_) using a UV–visible spectrophotometer. Untreated samples served as the control. All experiments were performed in triplicate (n = 3).

#### 2.5.4. Ultrastructure of *Xoo* Cells

Ultrastructural alterations in *Xoo* cells following nanoparticle treatment were examined by TEM based on the method described in [37]. Briefly, *Xoo* cultures were incubated with CA-ZnO NPs at a 1× MIC in PSB medium for 24 h at 28 °C. After incubation, bacterial cells were harvested by centrifugation at 5000 rpm for 10 min, washed three times with phosphate-buffered saline (PBS, pH 7.4), and fixed with 2.5% (*v*/*v*) glutaraldehyde at 4 °C for 24 h. Post-fixation was performed using 1% osmium tetroxide (OsO_4_) at 4 °C for 1 h. Samples were dehydrated through a graded ethanol series (30–100%), infiltrated with epoxy resin (Spurr’s Low Viscosity Embedding Media Kit, EMS), and embedded in pure resin. Ultrathin sections (~70–90 nm) were prepared using a diamond knife on a Leica ultramicrotome, mounted onto copper grids, and examined by TEM (FEI TECNAI G2 20) operated at an accelerating voltage of 200 kV. Post-staining with heavy metals was deliberately omitted to avoid interference with nanoparticle visualization.

#### 2.5.5. Fluorescence Imaging for Live/Dead Assay and ROS Detection

The integrity of *Xoo* cell membranes and intracellular ROS generation following CA-ZnO NP treatment were evaluated by fluorescence microscopy. *Xoo* cultures (1 × 10^5^ CFU/mL) were treated with CA-ZnO NPs at 1× MIC for 24 h in PSB medium at 28 °C. After incubation, bacterial cells were collected by centrifugation at 5000 rpm for 10 min, washed three times with normal saline, and resuspended in normal saline prior to staining.

For membrane integrity analysis, the LIVE/DEAD™ BacLight™ Bacterial Viability Kit (Invitrogen, Waltham, MA, USA) was employed following the manufacturer’s instructions. SYTO 9, a green fluorescent dye, penetrates all bacterial membranes, whereas propidium iodide (PI), a red fluorescent dye, selectively enters cells with compromised membranes, displacing SYTO 9 upon binding to DNA. Live cells emitted green fluorescence (Ex/Em ~480/500 nm), whereas membrane-damaged or dead cells emitted red fluorescence (Ex/Em ~535/617 nm), providing a qualitative assessment of cell membrane damage [59]. Imaging was performed using a Leica Mica Widefield Live Cell Imaging System (Leica Microsystems, Wetzlar, Germany).

For intracellular ROS detection, the resuspended cells were incubated with 2′,7′-dichlorodihydrofluorescein diacetate (DCFH-DA) at a final concentration of 20 µM in PBS. Cells were incubated at 28 °C for 20 min in the dark, followed by three washes with PBS to remove excess dye. Oxidation of DCFH-DA by intracellular ROS yielded the fluorescent compound dichlorofluorescein (DCF), which was visualized as green fluorescence (Ex/Em ~495/529 nm) [59]. Fluorescence microscopy images were acquired using a Nikon ECLIPSE series fluorescence microscope (Nikon Instruments Inc., Tokyo, Japan).

#### 2.5.6. Nucleic Acid Leakage Assay

To assess membrane damage, the leakage of nucleic acids (DNA and RNA) from *Xoo* cells was quantified after treatment with CA-ZnO NPs. Mid-log-phase bacterial cultures were treated with CA-ZnO NPs at a final concentration of 1× MIC (8 µg/mL) and incubated for 24 h at 28 °C under static conditions. Untreated cultures were used as the control.

After incubation, cultures were centrifuged at 10,000 rpm for 5 min to remove intact cells and debris. The resulting supernatants were collected, and the concentrations of extracellular DNA and RNA were quantified using a Qubit™ fluorometer (Thermo Fisher Scientific, Waltham, MA, USA) with the Qubit™ dsDNA HS Assay Kit and Qubit™ RNA HS Assay Kit (Invitrogen, Thermo Fisher Scientific, Waltham, MA, USA), respectively, following the manufacturer’s instructions. All measurements were performed in triplicate (*n* = 3).

#### 2.5.7. Zinc Ion Release Analysis Using ICP-OES

To evaluate the release of Zn^2+^ ions from CA-ZnO NPs, the nanoparticles were suspended at the MIC (8 µg/mL) in either DI water or PSB medium and incubated at room temperature for 24 h without agitation. After incubation, the suspensions were centrifuged, and the resulting supernatants were collected for elemental analysis.

The quantification of dissolved Zn^2+^ ions was conducted using Inductively Coupled Plasma Optical Emission Spectroscopy (ICP-OES) on a PerkinElmer Avio 550 Max instrument (PerkinElmer, Waltham, MA, USA). For each sample, 25 mL of the supernatant was mixed with 1.25 mL of superpure nitric acid (HNO_3_; Reagecon Diagnostics Ltd., Shannon, County Clare, Ireland; Lot no. 3088) and digested in a water bath (Memmert, WNB 45, Schwabach, Germany) at 95 ± 5 °C. The digested solution was filtered and diluted to a final volume of 25 mL before analysis. Calibration was performed using a standard curve with Zn concentrations of 0.005, 0.01, 0.05, 0.1, 0.5, 1.0, 2.5, and 5.0 ppm. The test method was adapted from the in-house protocol TE-CH-126 based on the *Standard Methods for the Examination of Water and Wastewater*, 24th edition, 2023 (APHA, AWWA, WEF), Part 330 E and Part 3120 B [60].

#### 2.5.8. Biocompatibility of CA-ZnO NPs with Human Dermal Fibroblasts

The biocompatibility of CA-ZnO NPs was evaluated using primary adult human dermal fibroblasts (HDFs) obtained from ATCC^®^ (PCS-201-012™, American Type Culture Collection, Manassas, VA, USA). Cells were cultured in Dulbecco’s Modified Eagle Medium (DMEM; Cat# 11965-092), supplemented with 10% fetal bovine serum (FBS; Cat# 10270-106) and Pen Strep (100 U/mL penicillin and 100 µg/mL streptomycin; Cat# 15140-122) (all from Gibco, Thermo Fisher Scientific, Waltham, MA, USA), and maintained at 37 °C in a humidified 5% CO_2_ atmosphere [61]. Upon reaching approximately 80% confluency, cells were detached using 0.25% Trypsin-EDTA (Cat# 25200-072, Gibco, Waltham, MA, USA) and seeded into 96-well plates at 1 × 10^4^ cells per well. After 24 h of incubation, cells were treated with various concentrations of CA-ZnO NPs and incubated for either 24 or 48 h.

Cell viability was assessed using a modified MTT assay protocol based on Mosmann [62]. Briefly, MTT reagent (5 mg/mL in PBS) was mixed with DMEM at a 1:10 ratio, and 100 µL of this mixture was added to each well. The plates were incubated at 37 °C for 4 h. After incubation, the medium was aspirated, and the resulting formazan crystals were dissolved by adding 100 µL of dimethyl sulfoxide (DMSO). Absorbance was measured at 570 and 630 nm using a microplate reader (SpectraMax M5, Molecular Devices, San Jose, CA, USA). The corrected absorbance was obtained by subtracting the 630 nm background from the 570 nm reading. All experiments were performed in triplicate. Cell viability (%) was calculated using the following formula:

Cell Viability (%) = (Absorbance of treated cells/Absorbance of control cells) × 100

### 2.6. Statistical Analysis

All biological experiments were performed in triplicate unless otherwise specified. Data are presented as mean ± standard error (SE). Statistical analyses were performed using SPSS version 22 (IBM Corp., Armonk, NY, USA). One-way ANOVA followed by Duncan’s multiple range test (DMRT) was applied to evaluate differences among treatment groups in the HDF cell viability assay. For other experiments involving comparisons between two groups, such as Zn^2+^ ion release and nucleic acid leakage, a two-tailed independent-samples t-test was used. In all cases, a *p*-value of less than 0.05 was considered statistically significant.

## 3. Results

### 3.1. Phytochemical Screening of C. asiatica Leaf Extract

Qualitative phytochemical analysis of the aqueous extract revealed the presence of amino acids, carbohydrates, alkaloids, flavonoids, phenols, quinones, saponins, steroids, tannins, and terpenoids, whereas proteins, reducing sugars, anthraquinones, cardiac glycosides, and glycosides were absent (Table 1). These bioactive compounds may contribute to the reduction and stabilization of nanoparticles during green synthesis.

### 3.2. Antioxidant Capacity (DPPH Assay)

**Figure 2:**
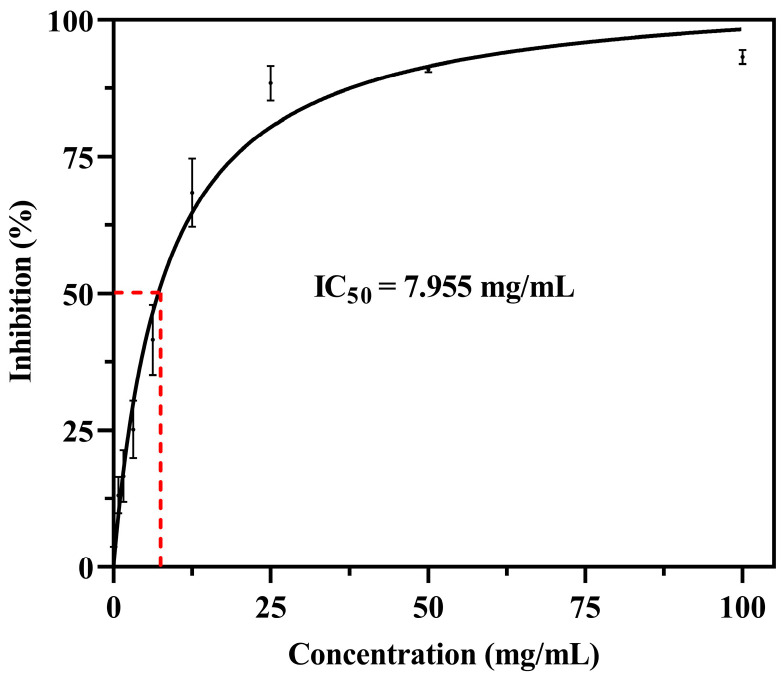
DPPH radical scavenging activity of *C. asiatica* leaf extract with dose-dependent inhibition. The red dashed vertical line indicates the IC_50_ value, determined as the concentration at which 50% of DPPH radical scavenging activity was observed.

The antioxidant activity of the *C. asiatica* leaf extract was evaluated by the DPPH radical scavenging assay. The extract exhibited dose-dependent scavenging activity, and the IC_50_ value was determined to be about 7.9 mg/mL (Figure 2).

### 3.3. Green Synthesis of CA-ZnO NPs

The formation of ZnO nanoparticles was achieved through a green synthesis route utilizing phytochemical-rich *C. asiatica* leaf extract. Visual observation of the reaction process revealed a gradual transformation to a pale yellow precipitate, indicating the formation of ZnO-based nanostructures. This color change was observed after adjusting the reaction mixture to alkaline pH and maintaining mild heating conditions (Figure 1), consistent with the conversion of Zn(OH)_2_ to ZnO under thermal treatment.

The as-prepared CA-ZnO NPs were subsequently collected, purified, and dried. These nanoparticles were then subjected to a series of characterization techniques, including UV–Vis spectroscopy, SEM, TEM, EDX, XRD, FTIR, and SAED, to confirm their structural, morphological, and compositional features.

### 3.4. Characterization of CA-ZnO NPs

#### 3.4.1. Optical Properties: UV–Vis Spectroscopy

**Figure 3:**
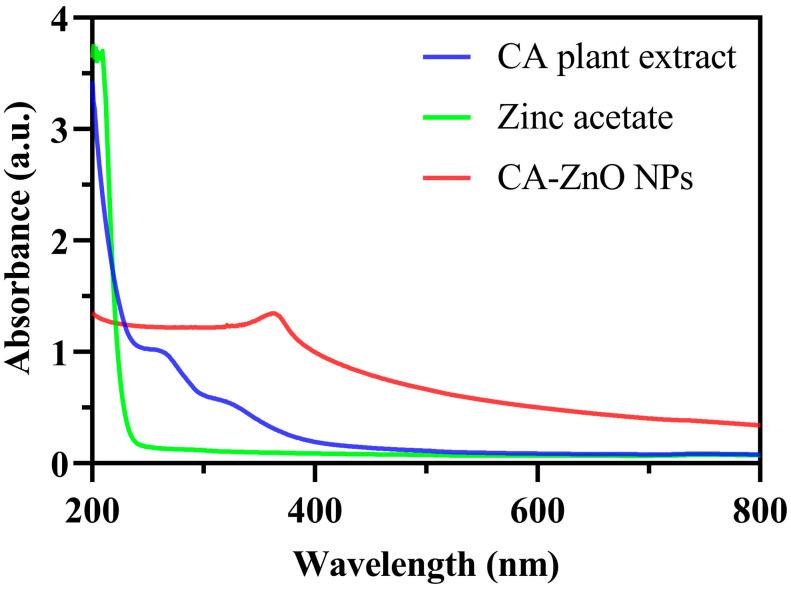
UV–Vis absorption spectra of CA-ZnO NPs (red line), *C. asiatica* (CA) leaf extract (blue line), and zinc acetate precursor (green line). The CA-ZnO NPs exhibit a distinct absorption peak at approximately 365 nm, which is not observed in the spectra of either the plant extract or the precursor.

The UV–Vis absorption spectra of zinc acetate, *C. asiatica* leaf extract, and CA-ZnO NPs are shown in Figure 3. The CA-ZnO NPs exhibited a prominent absorption peak at approximately 365 nm. In contrast, the zinc acetate precursor showed no significant absorption in the visible range, and the CA plant extract presented a broad absorption band below 350 nm. The absorption pattern of CA-ZnO NPs differed markedly from those of the precursor and the plant extract.

The optical band gap energy ($E_{g}$) was estimated using the Planck–Einstein relation:

$$E_{g}=\frac{hc}{\lambda }$$

where *h* is Planck’s constant (6.626 × 10^−34^ J·s), *c* is the speed of light (3 × 10^−8^ m/s), and *λ* is the maximum absorption wavelength (364 or 3.64 × 10^−7^). Substituting the values,

$$E_{g}=\frac{6.626\times 10^{-34}\times 3.0\times 10^{8}}{3.64\times 10^{-7}}=5.46\times 10^{-19}\;J$$

Converting to electron volts (1 eV = 1.602 × 10^−19^ J),

$$E_{g}=\frac{5.46\times 10^{-19}}{1.602\times 10^{-19}}\approx 3.41\;eV$$

Alternatively, the simplified equation $E_{g}\;\left(eV\right)=\frac{1240}{\lambda \;(nm)}$ gives the same results:

$$E_{g}=\frac{1240}{364}\approx 3.41\;eV$$

#### 3.4.2. Structural Properties: XRD and SAED Analysis

**Figure 4:**
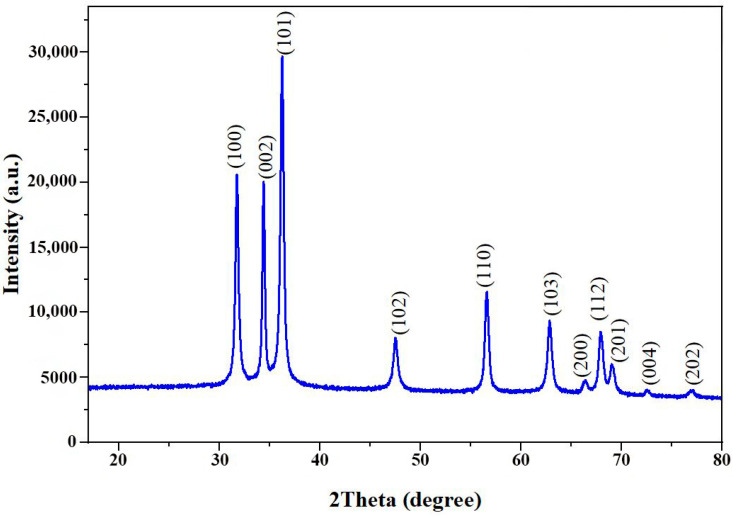
XRD pattern of CA-ZnO NPs confirming hexagonal wurtzite structure based on JCPDS card no. 36-1451.

The crystalline structure of the synthesized CA-ZnO NPs was characterized using XRD analysis. The diffraction pattern exhibited sharp and well-defined peaks at 2θ values of 31.74°, 34.39°, 36.23°, 47.51°, 56.58°, 62.83°, 66.37°, 67.93°, 69.04°, 72.54°, and 76.93°, corresponding to the (100), (002), (101), (102), (110), (103), (200), (112), (201), (004), and (202) planes of the hexagonal wurtzite ZnO crystal structure, as referenced by JCPDS card no. 36-1451 (Figure 4). The absence of additional peaks confirmed the phase purity of the product.

The average crystallite size (*D*) was calculated using the Scherrer equation:

*D* = *Kλ*/*β*cos*θ*

where *D* is the crystallite size (nm), *K* is the Scherrer constant (0.9), *λ* is the X-ray wavelength (1.5406 Å), *β* is the full width at half maximum (FWHM) in radians, and *θ* is the Bragg angle. The calculated average crystallite size was approximately 19.80 nm (Table 2).

**Figure 5:**
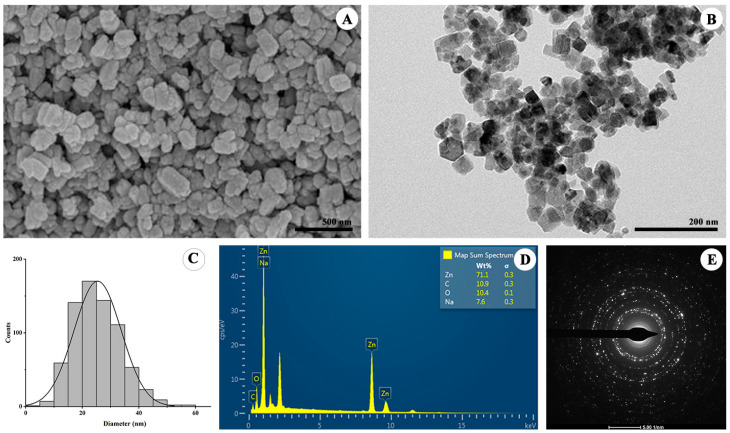
Morphological and structural characterization of CA-ZnO NPs: (**A**) SEM micrograph showing surface agglomeration, (**B**) TEM image showing quasi-spherical to hexagonal nanoparticles, (**C**) particle size distribution histogram from TEM, (**D**) EDX spectrum showing elemental composition, (**E**) SAED pattern indicating polycrystallinity.

Complementary structural analysis was performed by SAED. The SAED pattern of the CA-ZnO NPs displayed a series of concentric diffraction rings with discrete bright spots (Figure 5E), confirming the polycrystalline nature of the synthesized nanoparticles. The ring patterns matched the crystallographic planes identified in the XRD analysis, providing further validation of the hexagonal phase of ZnO.

#### 3.4.3. Morphological and Elemental Analysis

The FE-SEM images revealed agglomerated particles with rough and uneven surfaces (Figure 5A), a common characteristic of biosynthesized ZnO NPs due to the presence of surface-bound organic residues. TEM analysis provided detailed insights into particle shape and size, showing well-dispersed nanoparticles with quasi-spherical to hexagonal morphologies (Figure 5B). A particle size distribution histogram constructed from the TEM micrographs showed an average particle diameter of 22.5 ± 6.5 nm (Figure 5C), confirming nanoscale dimensions and relatively good size uniformity.

The elemental composition of the CA-ZnO NPs was further analyzed using the EDX technique. The EDX spectrum confirmed the presence of zinc (Zn) and oxygen (O) as the primary elements, consistent with the expected composition of ZnO. Prominent Zn peaks were observed at approximately 1.0, 8.6, and 9.6 keV, while the O peak appeared near 0.5 keV (Figure 5D). Additional minor peaks corresponding to carbon (C) and sodium (Na) were also detected. Quantitative analysis revealed that Zn was the dominant element (71.1 wt%), followed by O (10.4 wt%), C (10.9 wt%), and Na (7.6 wt%).

#### 3.4.4. Functional Group Analysis: FTIR Spectroscopy

**Figure 6:**
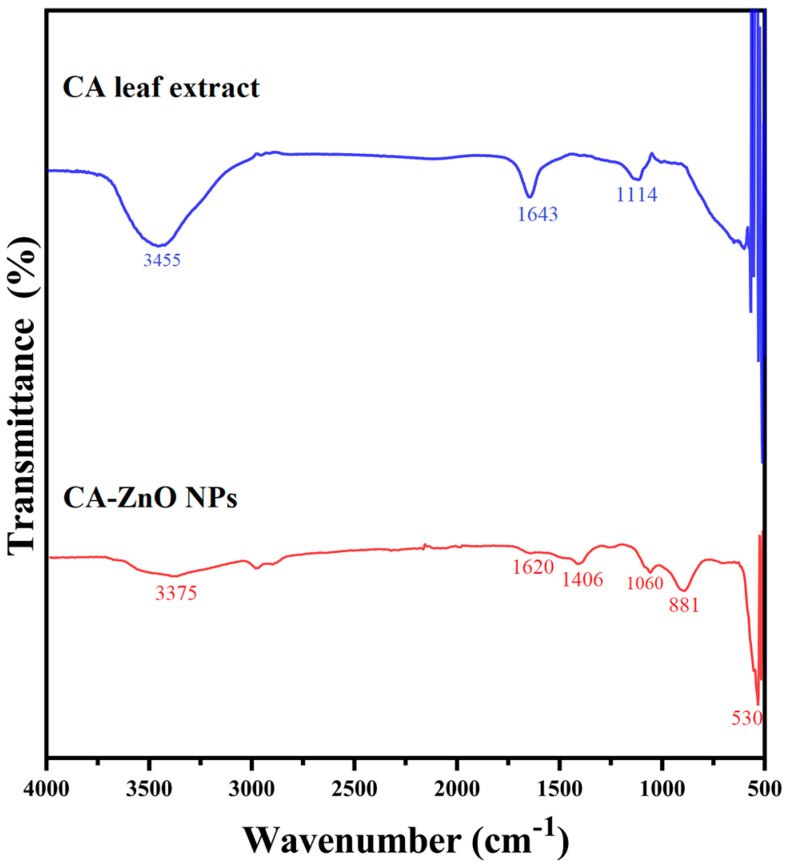
FTIR spectra of *C. asiatica* leaf extract and CA-ZnO NPs synthesized using the *C. asiatica* leaf extract.

The FTIR spectra of both *C. asiatica* (CA) leaf extract and CA-ZnO NPs are shown in Figure 6. For the CA extract, a broad absorption band was observed at 3455 cm^−1^, which corresponds to O–H stretching vibrations commonly found in alcohols and phenolic compounds. A peak at 1643 cm^−1^ was attributed to C=O stretching, indicating the presence of amide or conjugated carbonyl groups. Another prominent band at 1114 cm^−1^ is associated with C–O stretching vibrations of ether linkages or glycosidic bonds typically present in polyphenols or flavonoid glycosides.

In the spectrum of CA-ZnO NCs, a broad O–H stretching band appeared at 3375 cm^−1^, indicating a slight shift due to interaction with the ZnO nanoparticle surface. A medium-intensity peak at 1620 cm^−1^ was observed, which is attributed to C=O stretching (amide I region), also slightly shifted from the extract. A new peak at 1406 cm^−1^ was assigned to O–H bending or C–N stretching of amine-containing phytochemicals. Another band appeared at 1060 cm^−1^, attributed to C–O stretching, which was not clearly resolved in the extract spectrum. The absorption band at 881 cm^−1^ corresponds to out-of-plane C–H bending vibrations of aromatic compounds. A strong, sharp band at 530 cm^−1^ was observed, corresponding to Zn–O stretching vibrations and confirming the successful formation of ZnO nanoparticles.

#### 3.4.5. Size Distribution and Surface Charge: DLS and Zeta Potential Analysis

The hydrodynamic size distribution and surface charge of CA-ZnO NPs dispersed in DI water were evaluated using DLS and zeta potential measurements. The DLS profile exhibited a bimodal distribution, with a major peak at 176.7 ± 152.9 d.nm and a minor peak at 14.78 ± 2.53 d.nm, yielding a Z-average size of 98.94 d.nm and a PDI of 0.386, indicating moderate polydispersity (Figure S1). The zeta potential was recorded as −25.5 ± 8.87 mV (Figure S2), suggesting a moderate level of colloidal stability due to electrostatic repulsion.

### 3.5. Antibacterial Activity Against Xoo

#### 3.5.1. Minimum Inhibitory Concentration (MIC)

**Figure 7:**
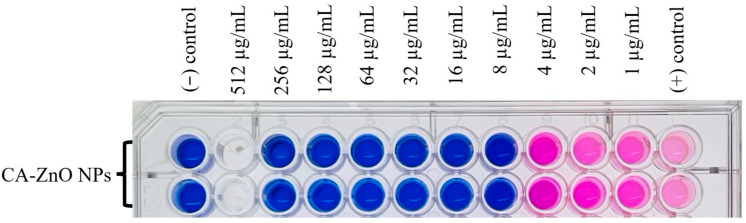
Colorimetric results of the resazurin-based MIC assay against *Xoo* showing concentration-dependent bacterial inhibition.

The MIC of CA-ZnO NPs against *Xoo*, determined by the broth microdilution method, was 8 µg/mL. A two-fold serial dilution was prepared over a concentration range of 512 µg/mL to 1 µg/mL. A concentration-dependent inhibition of bacterial growth was observed, as indicated by a resazurin color change from blue (viable cells) to pink (non-viable cells) (Figure 7).

#### 3.5.2. Agar Disk Diffusion Assay

**Figure 8:**
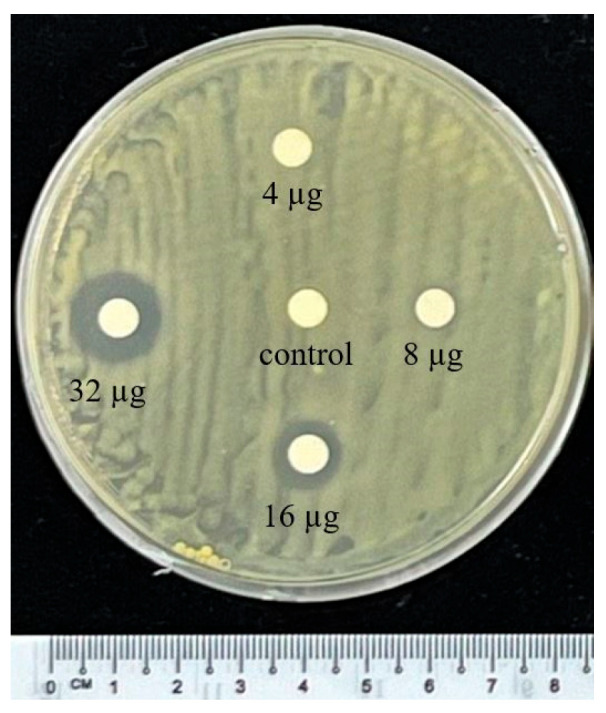
Zones of inhibition recorded from agar disk diffusion assay at different CA-ZnO NP concentrations (4, 8, 16, and 32 µg/disk) against *Xoo*.

In the disk diffusion assay, no zones of inhibition were observed at 4 or 8 µg/disk. At 16 µg/disk and 32 µg/disk, inhibition zones of 9.4 ± 0.4 mm and 11.5 ± 0.9 mm, respectively, were recorded (Figure 8, Table 3).

#### 3.5.3. Time–Kill Kinetics Assay

**Figure 9:**
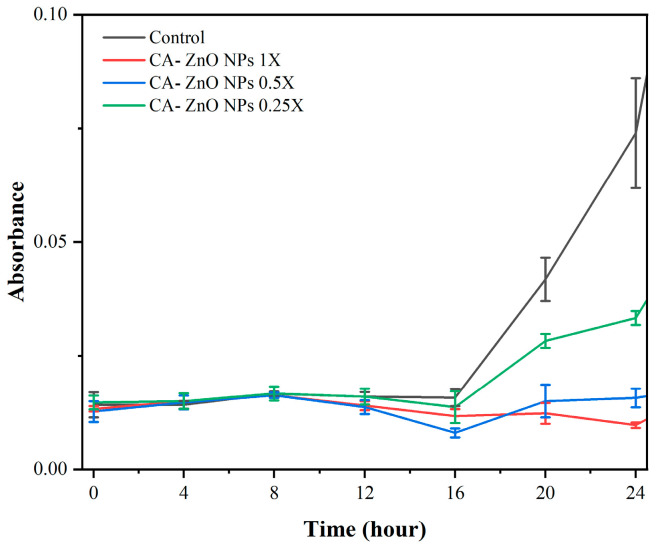
Time–kill kinetics of *Xoo* treated with CA-ZnO nanoparticles at 0.25×, 0.5×, and 1× MIC (8 µg/mL) over a 24 h incubation period.

The antibacterial activity of CA-ZnO NPs against *Xoo* was evaluated over a 24 h period by monitoring the optical density (OD_600_) of bacterial cultures treated with 0.25×, 0.5×, and 1× MICs. The MIC value was previously determined to be 8 µg/mL. At sub-inhibitory concentrations (0.25× and 0.5× MIC), bacterial growth continued over time, with OD_600_ values increasing in a pattern similar to the untreated control. In contrast, treatment with CA-ZnO NPs at 1× MIC (8 µg/mL) markedly suppressed bacterial growth, with a significant reduction in OD_600_ evident after 8 h and near-complete inhibition observed at 24 h (Figure 9). Based on these findings, the 1× MIC was selected for subsequent mechanistic studies due to its pronounced and sustained inhibitory effect.

### 3.6. Mechanism of Antibacterial Action

#### 3.6.1. Membrane Integrity Assessment by Live/Dead Assay

**Figure 10:**
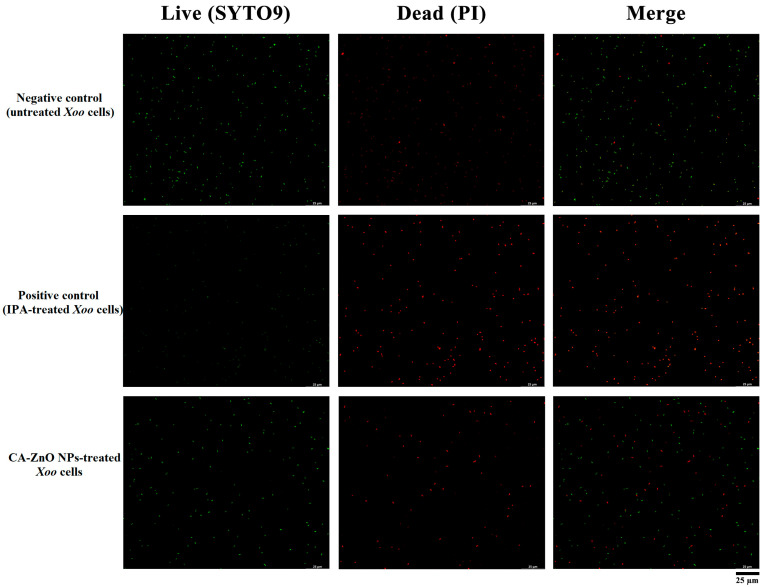
Widefield fluorescence images of *Xoo* cells stained with SYTO9 (green, live) and propidium iodide (red, dead) after 24 h treatment. Top panel: Negative control (untreated), showing primarily green fluorescence, indicating intact membranes. Middle panel: Positive control (70% isopropanol), displaying predominantly red fluorescence due to complete membrane disruption. Bottom panel: Cells treated with CA-ZnO NPs at 1× MIC, showing mixed green and red fluorescence, consistent with partial membrane damage and reduced viability. (Scale bar = 25 µm.)

The impact of CA-ZnO NPs on the membrane integrity of *Xoo* cells was assessed using a Live/Dead fluorescence assay. Fluorescence microscopy revealed that untreated *Xoo* cells predominantly emitted green fluorescence, indicative of intact and viable membranes (Figure 10). In contrast, cells treated with CA-ZnO NPs exhibited increased red fluorescence, suggesting compromised membrane integrity and reduced cell viability.

#### 3.6.2. Ultrastructural Observation by TEM

**Figure 11:**
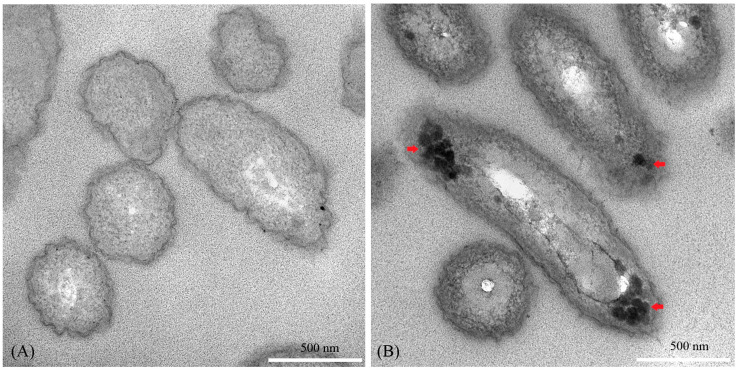
TEM images showing ultrastructural changes in *Xoo* cells. (**A**) Untreated control cells exhibit intact membranes and normal intracellular morphology. (**B**) Cells treated with CA-ZnO NPs at 1× MIC for 24 h show membrane disruption, cytoplasmic leakage, and internalized nanoparticles (indicated by red arrows). (Scale bar = 500 nm.)

Ultrastructural alterations in *Xoo* cells following CA-ZnO NP treatment were examined by TEM. Untreated control cells displayed intact cell envelopes, regular morphology, and uniformly distributed cytoplasmic content (Figure 11A). Conversely, CA-ZnO NP-treated cells exhibited severe membrane disintegration, cytoplasmic leakage, and visible internalization of nanoparticles within the cytoplasm (Figure 11B).

#### 3.6.3. Detection of Intracellular ROS Generation

**Figure 12:**
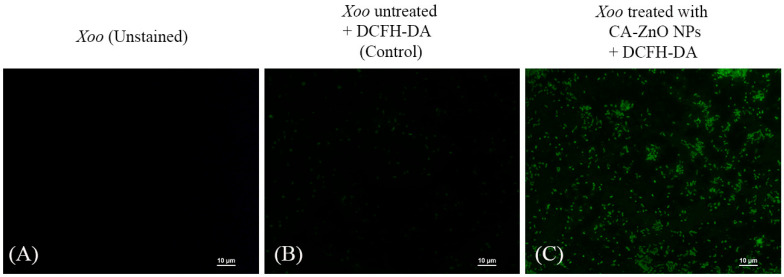
Fluorescence micrographs showing intracellular ROS generation in *Xoo* cells stained with the fluorescent probe DCFH-DA. (DCFH-DA. (**A**) *Xoo* (unstained); (**B**) *Xoo* untreated + DCFH-DA (Control); (**C**) *Xoo* treated with CA-ZnO NPs (1× MIC, 8 µg/mL, 24 h) + DCFH-DA. Increased green fluorescence in (**C**) indicates elevated ROS production in nanoparticle-treated cells. (Scale bar = 10 µm).

Intracellular ROS levels were evaluated using DCFH-DA staining. CA-ZnO NP-treated *Xoo* cells exhibited strong green fluorescence under fluorescence microscopy, indicating significant ROS accumulation (Figure 12). In comparison, untreated control cells showed only minimal background fluorescence. These results suggest that oxidative stress is involved in the antibacterial mechanism of CA-ZnO NPs.

#### 3.6.4. Nucleic Acid Leakage Assay

**Figure 13:**
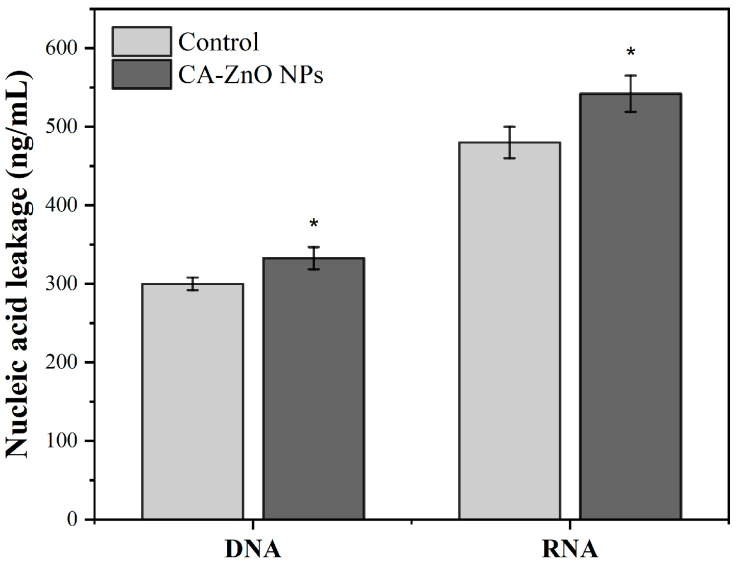
Leakage of DNA and RNA from *Xoo* cells after treatment with CA-ZnO NPs at 1× MIC (8 µg/mL) for 24 h. Nucleic acid concentrations in the extracellular medium were quantified using a Qubit fluorometer to assess membrane integrity. Data represent the mean ± standard deviation (SD) of three independent replicates (*n* = 3). Asterisks (*) indicate statistically significant differences compared to the corresponding control group (*p* < 0.05).

To assess membrane damage in *Xoo* cells upon exposure to CA-ZnO NPs, the release of intracellular nucleic acids (DNA and RNA) into the extracellular medium was quantified. As shown in Figure 13, treatment with CA-ZnO NPs at 1× MIC significantly increased the leakage of both DNA and RNA compared to the untreated control group. Specifically, DNA leakage increased from approximately 290 to 330 ng/mL, while RNA leakage rose from ~480 to 560 ng/mL. These results support the hypothesis that CA-ZnO NPs compromise bacterial membrane integrity, leading to nucleic acid efflux.

#### 3.6.5. Dissolution Behavior of Zn^2+^ Ions from CA-ZnO NPs in Deionized Water and PSB Medium

**Figure 14:**
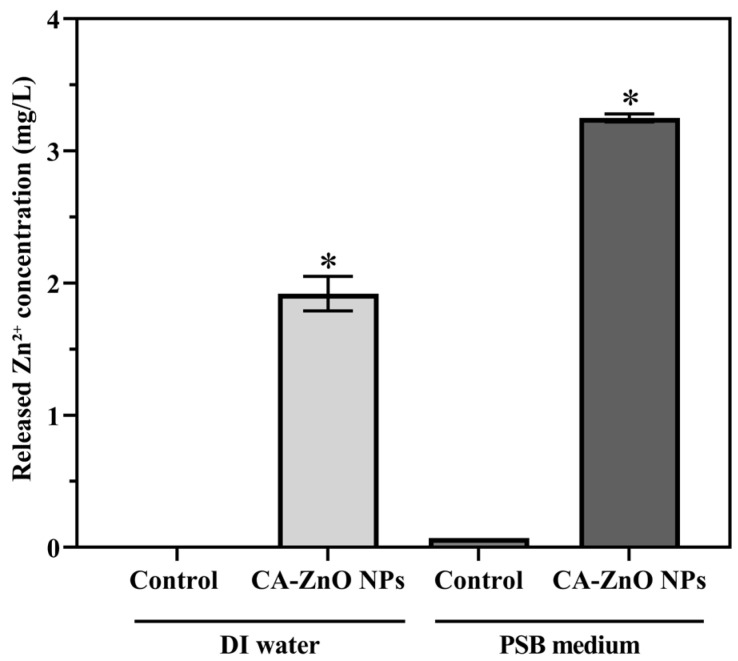
Concentration of Zn^2+^ ions released from CA-ZnO NPs (8 µg/mL) after 24 h incubation in deionized (DI) water and peptone sucrose broth (PSB) medium. Zinc ion concentrations in the supernatants were measured using ICP–OES. Data represent the mean ± standard deviation (SD) of three independent replicates (*n* = 3). Asterisks (*) indicate statistically significant differences compared to the corresponding control group (*p* < 0.05).

The release of Zn^2+^ ions from CA-ZnO NPs (8 µg/mL) was analyzed after 24 h of incubation in DI water and PSB medium using ICP-OES. In DI water, the Zn^2+^ concentration was 1.92 mg/L in the CA-ZnO NP-treated group, while no Zn^2+^ was detected in the control (0.00 mg/L). In PSB medium, the released Zn^2+^ concentration reached 3.25 mg/L in the presence of CA-ZnO NPs, compared to 0.07 mg/L in the control (Figure 14). These findings indicate that CA-ZnO NPs released measurable amounts of Zn^2+^ ions into both media during the 24 h incubation period, with higher dissolution observed in the PSB medium.

### 3.7. Biocompatibility Assessment on Human Dermal Fibroblasts (HDFs)

**Figure 15:**
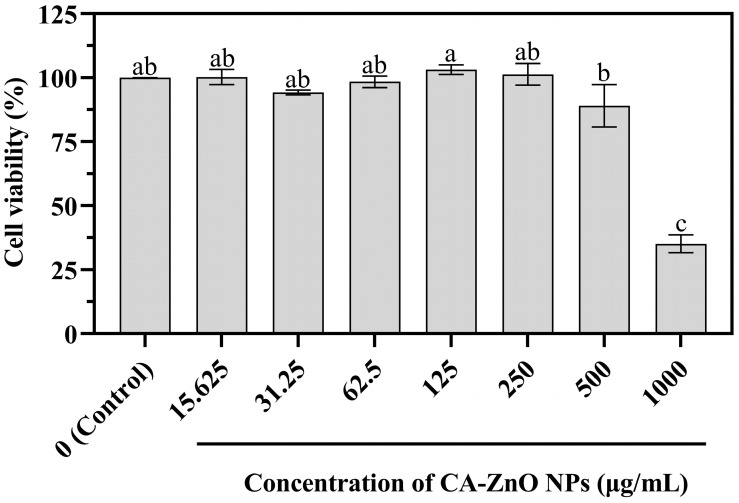
Cell viability of HDF cells after 24 h of exposure to various concentrations of CA-ZnO NPs, assessed using the MTT assay. Values are presented as mean ± SE (*n* = 3). Bars with the same letter are not significantly different according to Duncan’s multiple range test (*p* < 0.05).

The biocompatibility of CA-ZnO NPs was evaluated in HDFs using the MTT assay after 24 h of exposure. As shown in Figure 15, cell viability at all tested concentrations up to 500 µg/mL remained high and showed no statistically significant difference from the untreated control group (*p* > 0.05). A significant reduction in viability was observed only at 1000 µg/mL (*p* < 0.05), where viability decreased to approximately 35%. These results suggest that CA-ZnO NPs are biocompatible with human dermal fibroblasts at concentrations relevant for antimicrobial application.

## 4. Discussion

### 4.1. Green Synthesis and Characterization of CA-ZnO NPs

The successful formation of CA-ZnO NPs using *C. asiatica* leaf extract was confirmed by the immediate appearance of a pale yellow/white precipitate, consistent with typical green synthesis processes employing phytochemicals as stabilizing and functionalizing agents. Phytochemical screening revealed the presence of biomolecules such as flavonoids, phenols, and terpenoids, which are known to facilitate metal ion complexation and nanoparticle stabilization [63,64,65].

The formation of ZnO NPs was preliminarily checked by UV–visible spectroscopy measured in the range of 300–800 nm. The UV–Vis absorption peak at 365 nm observed for the CA-ZnO NPs corresponds well with the characteristic surface plasmon resonance (SPR) region of ZnO nanoparticles, which has been previously reported in the range of 360–380 nm [37,66,67]. Notably, the absorption maximum at 365 nm in the present study closely matches the findings of [68], who synthesized ZnO NPs using *Coriandrum sativum* leaf extract and zinc acetate precursor. In addition, the estimated band gap energy (Eg) of the synthesized CA-ZnO NPs was 3.41 eV, which is slightly higher than the reported value for bulk ZnO (∼3.3 eV at room temperature) [69]. This blueshift in the band gap can be attributed to the quantum confinement effect, whereby the reduction in particle size leads to the widening of the energy gap as the movement of charge carriers becomes more restricted [68]. The observed optical features thus strongly indicate the successful synthesis of ZnO NPs with nanoscale dimensions by the green synthesis route using *C. asiatica* extract.

The crystalline structure of the synthesized CA-ZnO NPs was characterized by XRD and SAED analyses. The XRD pattern exhibited multiple sharp and intense peaks at 2θ values of 31.74°, 34.39°, 36.23°, 47.51°, 56.58°, 62.83°, 66.37°, 67.93°, 69.04°, 72.54°, and 76.93°, which correspond to the (100), (002), (101), (102), (110), (103), (200), (112), (201), (004), and (202) planes, respectively. These diffraction peaks are in good agreement with the standard hexagonal wurtzite structure of ZnO, as referenced by JCPDS card No. 36-1451, confirming the phase purity and crystalline nature of the nanoparticles [24,70]. The sharpness and intensity of the peaks indicate high crystallinity, suggesting the formation of well-ordered crystalline structures with minimal defects [71]. Using the Scherrer equation, the average crystallite size was estimated to be approximately 19.80 nm, which is within the nanoscale range and comparable to previously reported biosynthesized ZnO NPs using *Phragmanthera austroarabica* and *Cocos nucifera* leaf extracts [66,72].

Complementing the XRD results, the SAED pattern obtained from TEM analysis displayed distinct concentric rings with bright diffraction spots, confirming the polycrystalline nature of the synthesized CA-ZnO nanoparticles. Notably, the diffraction rings corresponded well with the crystallographic planes identified in the XRD analysis, reaffirming the formation of hexagonal wurtzite ZnO. The consistency between XRD and SAED data provides strong evidence for the phase purity and excellent crystallinity of the biosynthesized ZnO nanoparticles.

Phytochemicals present in plant extracts are known to influence the nucleation kinetics and morphological evolution of metal oxide nanoparticles. Several studies have shown that these compounds can interact selectively with specific crystallographic planes during crystal growth, thereby promoting anisotropic development and modulating the final shape and size of the nanoparticles [73,74,75,76]. In this study, SEM and TEM analyses revealed that the synthesized CA-ZnO NPs exhibited quasi-spherical to hexagonal morphologies with an average particle size of 22.5 ± 6.5 nm. This dimension closely aligns with the crystallite size determined by XRD (~19.8 nm), suggesting the formation of single-crystalline or small-domain ZnO particles. The observed shapes and size uniformity may result from the modulatory effects of phytochemicals during synthesis, particularly through their role as capping and stabilizing agents, which help limit uncontrolled growth and particle aggregation.

The elemental composition of the synthesized ZnO NPs, as revealed by EDX analysis, further confirms the successful formation of ZnO. The strong signals for Zn and O are consistent with previous reports and support the phase purity of the nanoparticles [73,77]. The high Zn content, along with the presence of O, indicates the stoichiometric nature of ZnO, while the sharp Zn peaks suggest minimal impurity incorporation. In addition to Zn and O, trace amounts of carbon and sodium were also detected. The presence of these minor elements may be attributed to residual phytochemicals from the plant extract, which can remain on the nanoparticle surface as natural capping agents [70,72,77,78]. The sodium signal, despite repeated washing, may also originate from incomplete removal of NaOH used during the pH adjustment step. These minor components do not significantly affect the elemental purity of the ZnO NPs but rather suggest surface functionalization that is characteristic of green synthesis systems. Such surface-bound phytochemicals may enhance colloidal stability and promote biocompatibility for future applications.

FTIR analysis was used to identify the functional groups involved in the surface chemistry of CA-ZnO NPs. The spectrum of *C. asiatica* extract showed bands corresponding to O–H (3455 cm^−1^), C=O (1643 cm^−1^), and C–O (1114 cm^−1^) functional groups, commonly found in phenolics, flavonoids, and glycosides [23,46,47,79,80]. These phytochemicals are known to coordinate metal ions and adhere to nanoparticle surfaces through hydroxyl and carbonyl groups, contributing to stabilization rather than reduction [49,74].

In the CA-ZnO NPs spectrum, the O–H stretching peak shifted to 3375 cm^−1^, the C=O band to 1620 cm^−1^, and a new peak at 1060 cm^−1^ (C–O stretching) emerged more distinctly. These spectral shifts imply interactions between surface-bound phytochemicals and the ZnO structure, likely through hydrogen bonding or metal–ligand coordination [81,82]. Additional bands at 1406 and 881 cm^−1^ also support the presence of organic residues coating the nanoparticles. The sharp absorption at 530 cm^−1^, located within the Zn–O stretching region, confirms the successful formation of ZnO NPs [67,72]. These surface-bound functional groups, particularly hydroxyl and carbonyl moieties, are consistent with the presence of key phytochemicals identified through qualitative phytochemical screening of *C. asiatica* extract, including flavonoids, phenols, terpenoids, quinones, and tannins (Table 1). These bioactive compounds are known to bind metal oxide surfaces through –OH and C=O groups, serving as capping and stabilizing agents [46,47,49,74]. The FTIR peak shifts, such as the O–H stretching band shifting from 3455 to 3375 cm^−1^ and the C=O band from 1643 to 1620 cm^−1^, suggest that certain phytochemicals in the extract interact with Zn^2+^ ions through coordination or surface binding during the synthesis. Such interactions, rather than reduction, are consistent with previous reports in phytochemical-assisted nanoparticle systems [79,80]. These interactions contribute to the colloidal stabilization and surface functionalization of the CA-ZnO NPs.

To further understand the physical behavior of the nanoparticles in aqueous dispersion, DLS and zeta potential analyses were performed. The DLS profile revealed a bimodal distribution with a dominant hydrodynamic size around 176.7 ± 152.9 nm and a smaller population at 14.78 ± 2.53 nm, resulting in a Z-average size of 98.94 nm and a polydispersity index (PDI) of 0.386. The larger hydrodynamic diameter observed via DLS may be attributed to nanoparticle aggregation and hydration shells formed by surface-bound biomolecules, as similarly reported by [83].

The zeta potential of the CA-ZnO nanoparticle suspension was −25.5 ± 8.87 mV, indicating moderate colloidal stability [44,84]. This negative surface charge likely originates from anionic phytochemicals in *C. asiatica* extract, such as phenolics and triterpenoids, which adsorb onto the nanoparticle surface and confer electrostatic repulsion. While zeta potential provides useful insight, it does not fully predict dispersion stability. Other contributing forces, including van der Waals attraction and steric effects, also play important roles according to the DLVO theory [84]. Together, the moderate zeta potential and phytochemical capping suggest that CA-ZnO NPs are stabilized via electrosteric mechanisms in aqueous media.

### 4.2. Reevaluating the Bioreduction Hypothesis and Mechanism of CA-ZnO NPs Formation

Two main mechanisms have been proposed for the plant-mediated synthesis of ZnO nanoparticles. The first involves metal complexation between Zn^2+^ ions and phytochemicals, forming coordination complexes that decompose under thermal treatment to yield ZnO. The second mechanism suggests bioreduction of Zn^2+^ to elemental Zn^0^, followed by reoxidation to ZnO in the presence of dissolved oxygen. In both cases, phytochemicals are also believed to function as capping and stabilizing agents during nanoparticle formation [85,86].

Several studies have adopted the latter bioreduction-based model, suggesting that Zn^2+^ is reduced to Zn^0^ and then oxidized to ZnO [23,24,44,87,88,89]. This pathway has often been extrapolated from the well-established bioreduction of noble metals such as silver and gold [90,91]. However, such a mechanism is thermodynamically implausible under the mild aqueous conditions typically used in green synthesis.

The standard reduction potential (E^0^) for Zn^2+^/Zn^0^ is −0.76 V, significantly more negative than that of Ag^+^/Ag^0^ (+0.80 V) or Au^3+^/Au^0^ (+1.50 V) [92]. These noble metals can be readily reduced by plant-derived antioxidants, including flavonoids and polyphenols, whose redox potentials range from +0.4 to +0.7 V [93,94]. In contrast, the reduction of Zn^2+^ to Zn^0^ would require much stronger reducing agents, rendering such a mechanism unlikely under the mild aqueous conditions typically used in green synthesis. As such, a direct bioreduction route for ZnO nanoparticle synthesis appears chemically and thermodynamically unviable.

**Figure 16:**
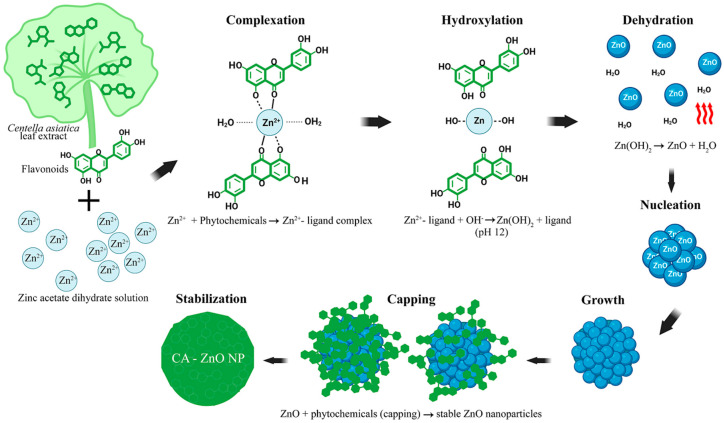
Proposed mechanistic pathway for the green synthesis of CA-ZnO NPs, highlighting sequential steps including complexation, hydroxylation, dehydration, nucleation, growth, capping, and stabilization mediated by phytochemicals, particularly flavonoids, present in *C. asiatica* extract. Thick arrows represent the sequential flow of steps in the proposed mechanism.

A more plausible mechanism for ZnO nanoparticle formation involves a multi-step process beginning with the complexation of Zn^2+^ ions with phytochemical ligands containing –OH, C=O, and –COOH functional groups [63,86]. Upon alkaline pH adjustment with NaOH, hydroxylation occurs, forming Zn(OH)_2_. This intermediate then undergoes hydration and nucleation under mild heating, followed by crystal growth and surface stabilization. The capping of ZnO nanoparticles by residual phytochemicals helps prevent aggregation and controls particle size and shape [73,74]. This refined mechanism can be summarized in five coordinated steps (Table 4 and Figure 16).

Understanding this corrected pathway supports the rational design of green synthesis protocols with better control over nanoparticle morphology, crystallinity, and biocompatibility.

### 4.3. Antibacterial Activity of CA-ZnO NPs Compared with Other Metal-Based Nanoparticles

The CA-ZnO NPs synthesized using *C. asiatica* extract demonstrated potent antibacterial activity against *Xoo*, a virulent Thai isolate NY1-1. The MIC was determined to be 8 µg/mL, which is among the lowest reported for biogenic ZnO NPs. To contextualize this performance, a comparative analysis of previously published ZnO NPs synthesized via green routes is presented in Table 5, summarizing particle size, morphology, synthesis method, and MIC values against *Xoo*.

As shown in Table 5, most ZnO nanoparticles synthesized using plant extracts, such as those derived from chamomile flower, olive leaves, red tomato fruit, mangosteen peel, and microbial extracts, exhibit MIC values ranging from 16 to 4000 µg/mL [37,39,41,95,96]. In contrast, CA-ZnO NPs—with an average size of approximately 22.5 nm and a morphology ranging from near-spherical to hexagonal—achieved a lower MIC, indicating superior antibacterial efficacy. Previous studies have shown that smaller ZnO NPs, due to their increased surface area to volume ratio, tend to interact more effectively with bacterial membranes and promote higher levels of ROS generation [25,26,97]. Conversely, ZnO NPs larger than 50 nm or those with irregular shapes, such as rods or aggregates, often require higher concentrations to achieve comparable antibacterial effects [41,96].

To ensure clarity in comparison, this analysis focuses solely on ZnO-based nanomaterials, thereby isolating the influence of particle size, shape, and synthetic route without the confounding effects of differing elemental compositions. While nanoparticles of silver (Ag) and copper (Cu) have demonstrated lower MIC values (e.g., 1.5–2.5 µg/mL) [98,99], their enhanced antimicrobial effects are largely attributed to the intrinsic redox properties and higher oxidative reactivity of these metals [100]. Therefore, Ag and Cu NPs serve as useful reference points for potency but are not directly comparable when assessing morphology-dependent performance of ZnO NPs.

In addition, a time–kill kinetics assay was employed to investigate the temporal dynamics of CA-ZnO NPs activity. At 1× MIC (8 µg/mL), a substantial suppression of bacterial growth was observed over 24 h, while treatments with 0.25× and 0.5× MIC resulted in only partial inhibition. These findings underscore the concentration-dependent nature of the antibacterial effect and further justify the selection of 1× MIC as the representative condition for mechanistic studies.

Taken together, the findings indicate that CA-ZnO NPs outperform most reported green-synthesized ZnO NPs in terms of antibacterial potency, attributable to their optimized nanoscale dimensions, uniform morphology, and effective phytochemical capping derived from *C. asiatica*. These features highlight the promise of CA-ZnO NPs for plant disease management, particularly in environmentally sensitive agricultural applications where green chemistry and biocompatibility are critical.

### 4.4. Mechanism of Antibacterial Action of ZnO NPs

ZnO NPs exhibit their antibacterial effects through several interrelated pathways, which include physical interaction with the bacterial membrane, penetration into the cytoplasmic space, the release of Zn^2+^ ions, and intracellular ROS generation. These mechanisms operate in concert to induce structural damage and functional impairment, ultimately leading to bacterial cell death [22,25,26].

In the present study, the Live/Dead fluorescence assay revealed a clear transition from green to red fluorescence in *Xoo* cells upon treatment with CA-ZnO NPs, indicating loss of membrane integrity and reduced cell viability. TEM observations further supported this finding, showing pronounced disruption of the cell envelope, leakage of intracellular material, and the presence of NPs within the cytoplasm. This suggests that ZnO NPs not only interact with the cell surface but also penetrate into the bacterial interior, interfering with homeostatic regulation.

A central feature of ZnO NP-mediated toxicity is the induction of ROS within bacterial cells. ROS such as superoxide anions (O_2_^−^), hydroxyl radicals (•OH), and hydrogen peroxide (H_2_O_2_) can damage cellular macromolecules, including lipids, proteins, and DNA, thereby disrupting homeostasis [26,101,102]. In our study, intracellular ROS generation was assessed using the DCFH-DA fluorescent probe under a fluorescence microscope. Although qualitative in nature, this approach is widely accepted in nanoparticle studies and has been successfully employed in previous reports [39,103]. Our findings revealed a marked increase in green fluorescence intensity in CA-ZnO NP-treated cells, supporting the role of oxidative stress in bacterial killing.

To further investigate the antibacterial mechanism, a time–kill kinetics assay was performed. While the primary objective was to assess efficacy (discussed in Section 4.3), the progressive suppression of *Xoo* growth over 24 h at 1× MIC suggests a cumulative mode of action consistent with oxidative and membrane-targeting pathways [25,37,97]. Supporting this interpretation, a nucleic acid leakage assay showed elevated levels of extracellular DNA and RNA following treatment with CA-ZnO NPs at 1× MIC. This result aligns with the fluorescence and TEM observations and implies substantial membrane disruption, allowing cytoplasmic content to escape. Similar phenomena have been documented in bacterial cells treated with ZnO NPs synthesized via other green routes [37,38,39].

Beyond physical and oxidative damage, Zn^2+^ ions released from the surface of ZnO NPs can further contribute to antibacterial activity. These ions interfere with bacterial metabolism by displacing essential metal cofactors, impairing ATP synthesis, disrupting membrane transport, and inhibiting nucleic acid transcription [97,104]. In this work, we quantified Zn^2+^ release from CA-ZnO NPs using ICP-OES. After 24 h of incubation, significant levels of Zn^2+^ were detected (1.92 ± 0.13 mg/L in deionized water and 3.25 ± 0.03 mg/L in PSB medium), while control samples showed negligible amounts. These results confirm that the nanoparticles release Zn^2+^ under both abiotic and biologically relevant conditions, supporting their role in metal ion-mediated toxicity.

These multifaceted antibacterial effects are consistent with the classic work by [105], who demonstrated that ZnO NPs exert toxicity toward *Escherichia coli* via a combination of membrane damage, oxidative stress, and ion dissolution. Their study further emphasized that the composition of the surrounding medium influences the extent of Zn^2+^ release and ROS generation, highlighting the complex interplay of physicochemical and biological factors governing ZnO NP-induced bacterial killing.

In addition to the primary mechanisms of ROS generation and membrane damage, an additional factor that may contribute to the antibacterial activity of CA-ZnO NPs is the presence of bioactive phytochemicals on the nanoparticle surface. *C. asiatica* has been shown to contain flavonoids and phenolic compounds with inherent antibacterial properties, including membrane-permeabilizing and ROS-enhancing activities [106]. These phytochemicals were likely retained during nanoparticle synthesis, as evidenced by FTIR analysis, which revealed functional groups consistent with hydroxyl and aromatic rings associated with flavonoids. Although the antibacterial effect of the extract alone was not evaluated in this study, previous reports indicate that flavonoids can destabilize bacterial membranes, inhibit metabolic enzymes, and facilitate oxidative damage [107]. Their interaction with bacterial surfaces may increase permeability, promote ZnO NP entry, and enhance localized ROS accumulation, thereby potentially amplifying the overall bactericidal effect in a synergistic manner.

**Figure 17:**
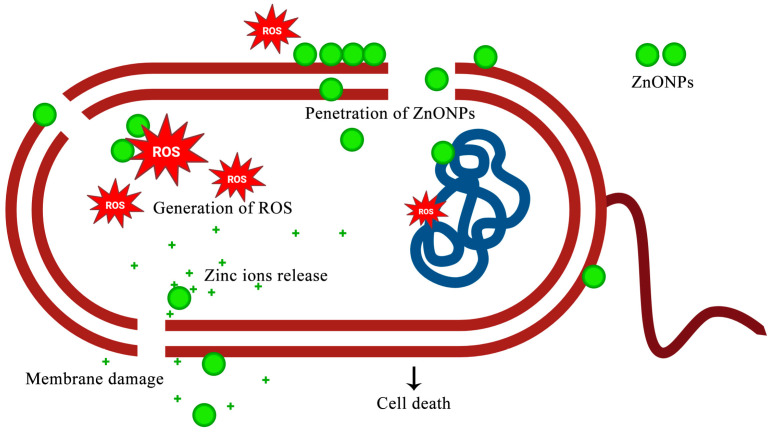
Proposed mechanism of antibacterial action of CA-ZnO NPs against *Xoo*. The nanoparticles interact with and disrupt the bacterial membrane, penetrate the cytoplasmic region, release Zn^2+^ ions, and induce ROS generation, resulting in molecular damage and bacterial cell death.

Figure 17 summarizes the proposed mechanism of action for CA-ZnO NPs. The particles adsorb onto the bacterial surface, induce membrane destabilization, penetrate the cytoplasm, release Zn^2+^, and generate ROS. These converging pathways compromise essential cellular functions and promote effective bacterial killing.

### 4.5. Biocompatibility in Human Cells

The biocompatibility of CA-ZnO NPs was assessed using human dermal fibroblasts (HDFs) to evaluate potential cytotoxic effects on non-target mammalian cells. Cell viability remained above 90% at concentrations up to 250 µg/mL, with a noticeable decline observed only at concentrations of 500 µg/mL and higher. These findings suggest that CA-ZnO NPs exhibit minimal cytotoxicity at concentrations well above the MIC of 8 µg/mL, supporting a favorable safety margin for biological applications.

In our study, CA-ZnO NPs exhibited no cytotoxicity toward HDF cells at MIC and sub-MIC levels, further supporting their biocompatibility and potential for biomedical applications. These findings are consistent with previous reports. Kaushik et al. [108] demonstrated that ZnO NPs promoted NIH3T3 fibroblast adhesion and proliferation, especially at larger particle sizes, while preserving normal morphology, membrane integrity, and F-actin organization [109] further showed that green-synthesized ZnO NPs enhanced in vitro wound closure by stimulating fibroblast migration. Similarly, Selim et al. [110] observed improved fibroblast proliferation and scratch wound healing following ZnO NP treatment, highlighting their potential role in tissue regeneration.

Although the antioxidant activity of the *C. asiatica* extract used in this study was classified as mild (DPPH IC_50_ ≈ 7.96 mg/mL), the aqueous extraction method is non-toxic and preserves phytochemicals with established biological relevance. Triterpenoids, saponins, and phenolic compounds, such as asiaticoside, madecassoside, and chlorogenic acid, which are commonly found in *C. asiatica*, have been shown to regulate intracellular reactive oxygen species, enhance antioxidant enzyme activity, and prevent apoptosis and senescence in mammalian cells [47,111]. It is plausible that some of these bioactive constituents remain associated with the nanoparticle surface during green synthesis and contribute synergistically to the biocompatibility of CA-ZnO NPs. This interpretation is further supported by FTIR data, which revealed O–H and C=O stretching vibrations that are typically attributed to phenolic and flavonoid compounds.

## 5. Conclusions

This study demonstrates a green, efficient, and biocompatible approach for the synthesis of ZnO NPs using *C. asiatica* leaf extract. The synthesized CA-ZnO NPs exhibited well-defined nanoscale dimensions, high crystallinity, and characteristic quasi-spherical to hexagonal morphology. Based on thermodynamic reasoning and the existing literature, the synthesis mechanism is more plausibly explained by a complexation–hydroxylation–nucleation–growth sequence, rather than by a bioreduction pathway often inferred from noble metal systems. Antibacterial assays showed that CA-ZnO NPs exhibited potent inhibitory effects against a highly virulent field isolate of *Xoo*, with a notably low MIC of 8 µg/mL. Mechanistic insights further confirmed multiple bactericidal actions, including membrane disruption, nanoparticle internalization, and ROS generation. Importantly, cytotoxicity assays on human dermal fibroblasts revealed excellent biocompatibility, with no statistically significant reduction in cell viability at concentrations up to 500 µg/mL. In contrast, a pronounced decrease in viability was observed at 1000 µg/mL. Given that the antibacterial MIC is more than 60-fold lower than the cytotoxic threshold, the CA-ZnO NPs offer a favorable therapeutic index and selective antimicrobial effect with minimal impact on mammalian cells. This dual functionality, which combines strong antimicrobial activity against plant pathogens with good compatibility toward mammalian cells, highlights the potential of CA-ZnO NPs as promising candidates for sustainable agricultural applications. They are particularly suited for the eco-friendly management of bacterial leaf blight in rice cultivation. Future studies should investigate their in-field efficacy, optimized formulation strategies, and long-term biosafety for broader implementation.

## Figures and Tables

**Table 1:** Qualitative phytochemical screening of the aqueous leaf extract of *C. asiatica*. (+: present; −: absent).

Phytoconstituents	Name of Detection Test/Reagent	Inference
**Biomolecules**		
Amino acids	Ninhydrin reagent	+
Carbohydrates	Anthrone reagent	+
Proteins	Biuret test	−
Reducing sugars	Fehling’s test	−
**Phytochemicals**		
Alkaloids	Mayer’s test	+
Anthraquinone	Ammonium solution	−
Cardiac glycoside	Keller–Kiliani test	−
Flavonoids	Alkaline reagent/Shinoda test	+
Glycoside	Ammonium solution	−
Phenol	Ferric chloride	+
Quinone	Sulphuric acid test	+
Saponin	Foam/Froth test	+
Steroids	Salkowski test	+
Tannins	Ferric chloride	+
Terpenoids	Salkowski’s test	+

**Table 2:** Crystallographic data and average crystallite size of CA-ZnO NPs as determined by the Scherrer equation.

Peak Number	Peak Position 2*θ* (°)	FWHM *β* (°)	Particle Size (*D*) [nm]	Average Crystallite Size (nm)
1	31.74	0.3687	22.40	19.80
2	34.39	0.2939	28.30	
3	36.23	0.3943	21.20	
4	47.51	0.5347	16.23	
5	56.58	0.4615	19.55	
6	62.83	0.5079	18.33	
7	66.37	0.5526	17.18	
8	67.93	0.4852	19.74	
9	69.04	0.5409	17.83	
10	72.54	0.4938	19.95	
11	76.93	0.5923	17.13	

**Table 3:** Inhibition zones of CA-ZnO NPs against *Xoo* determined by disk diffusion assay.

Treatment	Zone of Inhibition (ZOI) (mm)
Control	0
4 µg/disk	0
8 µg/disk	0
16 µg/disk	9.4 ± 0.4
32 µg/disk	11.5 ± 0.9

**Table 4:** Proposed multi-step mechanism for the green synthesis of CA-ZnO NPs, illustrating sequential events from metal–phytochemical complexation to nanoparticle stabilization.

Step	Event	Representative Reaction
1	Complexation	Zn^2+^ + phytochemicals → Zn^2+^ − ligand complex
2	Hydroxylation	Zn^2+^ − ligand + OH^−^ → Zn(OH)_2_ + ligands
3	Dehydration/Nucleation	Zn(OH)_2_ (heat, aqueous) → ZnO nuclei + H_2_O
4	Growth	Aggregation and directional growth of ZnO nanocrystals
5	Capping and Stabilization	ZnO + phytochemicals → surface-capped, stabilized NPs

**Table 5:** Comparative physicochemical characteristics and antibacterial activity (MIC) of green-synthesized ZnO NPs against *Xoo*.

No.	Biological Source	Size (nm)	Shape	MIC (µg/mL)	Reference
1	*Centella asiatica* leaves	22.5 ± 6.5	Hexagonal/quasi-spherical	8	This study
2	Chamomile flower	41.0 ± 2.0	Cubic	16	[37]
3	Olive leaves	51.2 ± 3.2	Cubic	16	[37]
4	Red tomato fruit	51.6 ± 3.6	Cubic	16	[37]
5	Rhizophytic bacteria *Paenibacillus polymyxa* strain Sx3	56.1–110	Cubic	16	[39]
6	Mangosteen peel	321 ± 84	Spherical	4000	[41]
7	*Trichoderma* spp.	12–35	Hexagonal	25–50	[95]
8	*Fusarium solani*	117.8–175.1	Irregular/nanorod-like	256–512	[96]

## Data Availability

The data presented in this study are available in this article and Supplementary Materials. Further inquiries can be directed to the corresponding authors.

## Supplementary Materials

The following supporting information can be downloaded at: https://www.mdpi.com/article/10.3390/nano15131011/s1, Figure S1: Dynamic light scattering (DLS) size distribution of CA-ZnO NPs dispersed in deionized water. Figure S2: Zeta potential measurement of CA-ZnO NPs in deionized water.

## References

[1] Jain L., Kumar V., Jain S.K., Kaushal P., Ghosh P.K. (2023). Isolation of bacteriophages infecting *Xanthomonas oryzae* pv. *oryzae* and genomic characterization of novel phage vB_XooS_NR08 for biocontrol of bacterial leaf blight of rice. Front. Microbiol..

[2] Kumar A., Kumar R., Sengupta D., Das S.N., Pandey M.K., Bohra A., Sharma N.K., Sinha P., Sk H., Ghazi I.A. (2020). Deployment of genetic and genomic tools toward gaining a better understanding of rice-*Xanthomonas oryzae* pv. *oryzae* interactions for development of durable bacterial blight resistant rice. Front. Plant Sci..

[3] Ritbamrung O., Inthima P., Ratanasut K., Sujipuli K., Rungrat T., Buddhachat K. (2025). Evaluating *Xanthomonas oryzae* pv. *oryzae* (*Xoo*) infection dynamics in rice for distribution routes and environmental reservoirs by molecular approaches. Sci. Rep..

[4] Mansfield J., Genin S., Magori S., Citovsky V., Sriariyanum M., Ronald P., Dow M., Verdier V., Beer S.V., Machado M.A. (2012). Top 10 plant pathogenic bacteria in molecular plant pathology. Mol. Plant Pathol..

[5] An S.-Q., Potnis N., Dow M., Vorhölter F.-J., He Y.-Q., Becker A., Teper D., Li Y., Wang N., Bleris L. (2020). Mechanistic insights into host adaptation, virulence and epidemiology of the phytopathogen *Xanthomonas*. FEMS Microbiol. Rev..

[6] Song Z., Zheng J., Zhao Y., Yin J., Zheng D., Hu H., Liu H., Sun M., Ruan L., Liu F. (2023). Population genomics and pathotypic evaluation of the bacterial leaf blight pathogen of rice reveals rapid evolutionary dynamics of a plant pathogen. Front. Cell. Infect. Microbiol..

[7] Zhang H., Wang S. (2013). Rice versus *Xanthomonas oryzae* pv. *oryzae*: A unique pathosystem. Curr. Opin. Plant Biol..

[8] Jiang N., Yan J., Liang Y., Shi Y., He Z., Wu Y., Zeng Q., Liu X., Peng J. (2020). Resistance genes and their interactions with bacterial blight/leaf streak pathogens (*Xanthomonas oryzae*) in rice (*Oryza sativa* L.)—An updated review. Rice.

[9] Niño-Liu D.O., Ronald P.C., Bogdanove A.J. (2006). *Xanthomonas oryzae* pathovars: Model pathogens of a model crop. Mol. Plant Pathol..

[10] Xu X., Li Y., Xu Z., Yan J., Wang Y., Wang Y., Cheng G., Zou L., Chen G. (2022). TALE-induced immunity against the bacterial blight pathogen *Xanthomonas oryzae* pv. *oryzae* in rice. Phytopathol. Res..

[11] Teja B.S., Jamwal G., Gupta V., Verma M., Sharma A., Sharma A., Pandit V. (2025). Biological control of bacterial leaf blight (BLB) in rice–A sustainable approach. Heliyon.

[12] Adedibu P.A., Son O., Tekutyeva L., Balabanova L. (2024). Pathogenomic insights into *Xanthomonas oryzae* pv. *oryzae*’s resistome, virulome, and diversity for improved rice blight management. Life.

[13] Sahu S.K., Zheng P., Yao N. (2018). Niclosamide blocks rice leaf blight by inhibiting biofilm formation of *Xanthomonas oryzae*. Front. Plant Sci..

[14] McManus P.S., Stockwell V.O., Sundin G.W., Jones A.L. (2002). Antibiotic use in plant agriculture. Annu. Rev. Phytopathol..

[15] Sundin G.W., Wang N. (2018). Antibiotic resistance in plant-pathogenic bacteria. Annu. Rev. Phytopathol..

[16] Guan Y., Shen P., Lin M., Ye X. (2022). Exogenous alanine reverses the bacterial resistance to Zhongshengmycin with the promotion of the P cycle in *Xanthomonas oryzae*. Antibiotics.

[17] Kim S.-I., Song J.T., Jeong J.-Y., Seo H.S. (2016). Niclosamide inhibits leaf blight caused by *Xanthomonas oryzae* in rice. Sci. Rep..

[18] Daniel A.I., Keyster M., Klein A. (2023). Biogenic zinc oxide nanoparticles: A viable agricultural tool to control plant pathogenic fungi and its potential effects on soil and plants. Sci. Total Environ..

[19] Gupta A., Rayeen F., Mishra R., Tripathi M., Pathak N. (2023). Nanotechnology applications in sustainable agriculture: An emerging eco-friendly approach. Plant Nano Biol..

[20] Francis D.V., Abdalla A.K., Mahakham W., Sarmah A.K., Ahmed Z.F.R. (2024). Interaction of plants and metal nanoparticles: Exploring its molecular mechanisms for sustainable agriculture and crop improvement. Environ. Int..

[21] Dejene B.K. (2024). Biosynthesized ZnO nanoparticle-functionalized fabrics for antibacterial and biocompatibility evaluations in medical applications: A critical review. Mater. Today Chem..

[22] Mendes A.R., Granadeiro C.M., Leite A., Pereira E., Teixeira P., Poças F. (2024). Optimizing antimicrobial efficacy: Investigating the impact of zinc oxide nanoparticle shape and size. Nanomaterials.

[23] Naiel B., Fawzy M., Halmy M.W.A., Mahmoud A.E.D. (2022). Green synthesis of zinc oxide nanoparticles using Sea Lavender (*Limonium pruinosum* L. Chaz.) extract: Characterization, evaluation of anti-skin cancer, antimicrobial and antioxidant potentials. Sci. Rep..

[24] Naseer M., Aslam U., Khalid B., Chen B. (2020). Green route to synthesize zinc oxide nanoparticles using leaf extracts of *Cassia fistula* and *Melia azadarach* and their antibacterial potential. Sci. Rep..

[25] Sirelkhatim A., Mahmud S., Seeni A., Kaus N.H.M., Ann L.C., Bakhori S.K.M., Hasan H., Mohamad D. (2015). Review on zinc oxide nanoparticles: Antibacterial activity and toxicity mechanism. Nano-Micro Lett..

[26] Jiang S., Lin K., Cai M. (2020). ZnO nanomaterials: Current advancements in antibacterial mechanisms and applications. Front. Chem..

[27] Ashour M.A., Abd-Elhalim B.T. (2024). Biosynthesis and biocompatibility evaluation of zinc oxide nanoparticles prepared using *Priestia megaterium* bacteria. Sci. Rep..

[28] Khan R.A.A., Tang Y., Naz I., Alam S.S., Wang W., Ahmad M., Najeeb S., Rao C., Li Y., Xie B. (2021). Management of *Ralstonia solanacearum* in tomato using ZnO nanoparticles synthesized through *Matricaria chamomilla*. Plant Dis..

[29] Rehman F.U., Paker N.P., Khan M., Naeem M., Munis M.F.H., Rehman S.U., Chaudhary H.J. (2023). Bio-fabrication of zinc oxide nanoparticles from *Picea smithiana* and their potential antimicrobial activities against *Xanthomonas campestris* pv. *Vesicatoria* and *Ralstonia solanacearum* causing bacterial leaf spot and bacterial wilt in tomato. World J. Microbiol. Biotechnol..

[30] Parveen A., Siddiqui Z.A. (2021). Zinc oxide nanoparticles affect growth, photosynthetic pigments, proline content and bacterial and fungal diseases of tomato. Arch. Phytopathol. Plant Prot..

[31] González-Merino A.M., Hernández-Juárez A., Betancourt-Galindo R., Ochoa-Fuentes Y.M., Valdez-Aguilar L.A., Limón-Corona M.L. (2021). Antifungal activity of zinc oxide nanoparticles in *Fusarium oxysporum*-*Solanum lycopersicum* pathosystem under controlled conditions. J. Phytopathol..

[32] AlHarethi A.A., Abdullah Q.Y., AlJobory H.J., Anam A.M., Arafa R.A., Farroh K.Y. (2024). Zinc oxide and copper oxide nanoparticles as a potential solution for controlling *Phytophthora infestans*, the late blight disease of potatoes. Discov. Nano.

[33] Aftab Z.-H., Mirza F.S., Anjum T., Rizwana H., Akram W., Aftab M., Ali M.D., Li G. (2025). Antifungal potential of biogenic zinc oxide nanoparticles for controlling Cercospora leaf spot in mung bean. Nanomaterials.

[34] Kongtragoul P., Ahuja A.J. (2024). Fungicidal activity of some essential oils and zinc oxide nanoparticles on *Colletotrichum capsici* and *C. gloeosporioides* causing chili anthracnose. Acta Hortic..

[35] Mosquera-Sánchez L.P., Arciniegas-Grijalba P.A., Patiño-Portela M.C., Guerra–Sierra B.E., Muñoz-Florez J.E., Rodríguez-Páez J.E. (2020). Antifungal effect of zinc oxide nanoparticles (ZnO-NPs) on *Colletotrichum* sp., causal agent of anthracnose in coffee crops. Biocatal. Agric. Biotechnol..

[36] Cheema A.I., Ahmed T., Abbas A., Noman M., Zubair M., Shahid M. (2022). Antimicrobial activity of the biologically synthesized zinc oxide nanoparticles against important rice pathogens. Physiol. Mol. Biol. Plants.

[37] Ogunyemi S.O., Abdallah Y., Zhang M., Fouad H., Hong X., Ibrahim E., Masum M.M.I., Hossain A., Mo J., Li B. (2019). Green synthesis of zinc oxide nanoparticles using different plant extracts and their antibacterial activity against *Xanthomonas oryzae* pv. *oryzae*. Artif. Cells Nanomed. Biotechnol..

[38] Abdallah Y., Liu M., Ogunyemi S.O., Ahmed T., Fouad H., Abdelazez A., Yan C., Yang Y., Chen J., Li B. (2020). Bioinspired green synthesis of chitosan and zinc oxide nanoparticles with strong antibacterial activity against rice pathogen *Xanthomonas oryzae* pv. *oryzae*. Molecules.

[39] Ogunyemi S.O., Zhang M., Abdallah Y., Ahmed T., Qiu W., Ali M.A., Yan C., Yang Y., Chen J., Li B. (2020). The bio-synthesis of three metal oxide nanoparticles (ZnO, MnO_2_, and MgO) and their antibacterial activity against the bacterial leaf blight pathogen. Front. Microbiol..

[40] Parveen K., Kumar N., Ledwani L. (2022). Green synthesis of zinc oxide nanoparticles mediated from *Cassia renigera* bark and detect its effects on four varieties of rice. ChemistrySelect.

[41] Jaithon T., Atichakaro T., Phonphoem W., T-Thienprasert J., Sreewongchai T., T-Thienprasert N.P. (2024). Potential usage of biosynthesized zinc oxide nanoparticles from mangosteen peel ethanol extract to inhibit *Xanthomonas oryzae* and promote rice growth. Heliyon.

[42] Chumpol A., Monkham T., Saepaisan S., Sanitchon J., Falab S., Chankaew S. (2022). Phenotypic broad spectrum of bacterial blight disease resistance from Thai indigenous upland rice germplasms implies novel genetic resource for breeding program. Agronomy.

[43] Dey S., lochan Mohanty D., Divya N., Bakshi V., Mohanty A., Rath D., Das S., Mondal A., Roy S., Sabui R. (2025). A critical review on zinc oxide nanoparticles: Synthesis, properties and biomedical applications. Int. Pharm..

[44] Al-darwesh M.Y., Ibrahim S.S., Mohammed M.A. (2024). A review on plant extract mediated green synthesis of zinc oxide nanoparticles and their biomedical applications. Results Chem..

[45] Sugitha S.K.J., Venkatesan R., Latha R.G., Vetcher A.A., Al-Asbahi B.A., Kim S.-C. (2024). A study on the antibacterial, antispasmodic, antipyretic, and anti-inflammatory activity of ZnO nanoparticles using leaf extract from *Jasminum sambac* (L. Aiton). Molecules.

[46] Gray N.E., Alcazar Magana A., Lak P., Wright K.M., Quinn J., Stevens J.F., Maier C.S., Soumyanath A. (2018). *Centella asiatica*: Phytochemistry and mechanisms of neuroprotection and cognitive enhancement. Phytochem. Rev..

[47] Kunjumon R., Johnson A.J., Baby S. (2022). *Centella asiatica*: Secondary metabolites, biological activities and biomass sources. Phytomed. Plus.

[48] Pavan Kumar M.A., Suresh D., Sneharani A.H. (2021). *Centella asiatica* mediated facile green synthesis of nano zinc oxide and its photo-catalytic and biological properties. Inorg. Chem. Commun..

[49] Vipina Vinod T.N., Mathew A.S., Mathew D., Mathew J., Radhakrishnan E.K. (2025). Facile microwave-assisted green synthesis and characterization of flower shaped zinc oxide nanoclusters using *Centella asiatica* (Linn.) leaf extract and evaluation of its antimicrobial activity and in vivo toxic effects on *Artemia* nauplii. Prep. Biochem. Biotechnol..

[50] AOAC (2023). Guidelines for the Standardization of Botanical Ingredients.

[51] Mahakham W., Theerakulpisut P., Maensiri S., Phumying S., Sarmah A.K. (2016). Environmentally benign synthesis of phytochemicals-capped gold nanoparticles as nanopriming agent for promoting maize seed germination. Sci. Total Environ..

[52] Rahman M., Islam B., Biswas M., Khurshid Alam A.H.M. (2015). In vitro antioxidant and free radical scavenging activity of different parts of *Tabebuia pallida* growing in Bangladesh. BMC Res. Notes.

[53] Chandra H., Patel D., Kumari P., Jangwan J.S., Yadav S. (2019). Phyto-mediated synthesis of zinc oxide nanoparticles of *Berberis aristata*: Characterization, antioxidant activity and antibacterial activity with special reference to urinary tract pathogens. Mater. Sci. Eng. C.

[54] Gupta M., Tomar R.S., Kaushik S., Mishra R.K., Sharma D. (2018). Effective antimicrobial activity of green ZnO nano particles of *Catharanthus roseus*. Front. Microbiol..

[55] Ke Y., Hui S., Yuan M. (2017). *Xanthomonas oryzae* pv. *oryzae* inoculation and growth rate on rice by leaf clipping method. Bio-Protocol.

[56] Clinical and Laboratory Standards Institute (2020). Performance Standards for Antimicrobial Susceptibility Testing.

[57] Sarker S.D., Nahar L., Kumarasamy Y. (2007). Microtitre plate-based antibacterial assay incorporating resazurin as an indicator of cell growth, and its application in the in vitro antibacterial screening of phytochemicals. Methods.

[58] Hudzicki J. (2009). Kirby-Bauer Disk Diffusion Susceptibility Test Protocol.

[59] Ramalingam B., Parandhaman T., Das S.K. (2016). Antibacterial effects of biosynthesized silver nanoparticles on surface ultrastructure and nanomechanical properties of gram-negative bacteria viz. *Escherichia coli* and *Pseudomonas aeruginosa*. ACS Appl. Mater. Interfaces.

[60] American Public Health Association (APHA), American Water Works Association (AWWA), Water Environment Federation (WEF) (2023). Standard Methods for the Examination of Water and Wastewater.

[61] Freshney R.I. (2010). Culture of Animal Cells: A Manual of Basic Technique and Specialized Applications.

[62] Mosmann T. (1983). Rapid colorimetric assay for cellular growth and survival: Application to proliferation and cytotoxicity assays. J. Immunol. Methods.

[63] Król A., Railean-Plugaru V., Pomastowski P., Buszewski B. (2019). Phytochemical investigation of *Medicago sativa* L. extract and its potential as a safe source for the synthesis of ZnO nanoparticles: The proposed mechanism of formation and antimicrobial activity. Phytochem. Lett..

[64] Bhattacharjee N., Som I., Saha R., Mondal S. (2024). A critical review on novel eco-friendly green approach to synthesize zinc oxide nanoparticles for photocatalytic degradation of water pollutants. Int. J. Environ. Anal. Chem..

[65] Uysal Y., Görkem Doğaroğlu Z., Çaylali Z., Karakulak D.S. (2024). Rosemary-mediated green synthesis of ZnO nanoparticles and their integration into hydrogel matrices: Evaluating effects on wheat growth and antibacterial properties. Glob. Chall..

[66] Alahdal F.A.M., Qashqoosh M.T.A., Manea Y.K., Salem M.A.S., Khan A.M.T., Naqvi S. (2022). Eco-friendly synthesis of zinc oxide nanoparticles as nanosensor, nanocatalyst and antioxidant agent using leaf extract of *P. austroarabica*. OpenNano.

[67] Younas M.U., Iqbal M., Ahmad N., Iqbal S., Kausar A., Nazir A., Mohammed O.A., Anjum F. (2025). Biogenic synthesis of zinc oxide nanoparticles using NARC G1 garlic (*Allium sativum*) extract, their photocatalytic activity for dye degradation and antioxidant activity of the extract. Results Chem..

[68] Singh J., Kaur S., Kaur G., Basu S., Rawat M. (2019). Biogenic ZnO nanoparticles: A study of blueshift of optical band gap and photocatalytic degradation of reactive yellow 186 dye under direct sunlight. Green Process. Synth..

[69] Özgür Ü., Alivov Y.I., Liu C., Teke A., Reshchikov M.A., Doğan S., Avrutin V., Cho S.-J., Morkoç H. (2005). A comprehensive review of ZnO materials and devices. J. Appl. Phys..

[70] Rajendran N.K., George B.P., Houreld N.N., Abrahamse H. (2021). Synthesis of zinc oxide nanoparticles using *Rubus fairholmianus* root extract and their activity against pathogenic bacteria. Molecules.

[71] Bindu P., Thomas S. (2014). Estimation of lattice strain in ZnO nanoparticles: X-ray peak profile analysis. J. Theor. Appl. Phys..

[72] Rahman F., Majed Patwary M.A., Bakar Siddique M.A., Bashar M.S., Haque M.A., Akter B., Rashid R., Haque M.A., Royhan Uddin A.K.M. (2022). Green synthesis of zinc oxide nanoparticles using *Cocos nucifera* leaf extract: Characterization, antimicrobial, antioxidant and photocatalytic activity. R. Soc. Open Sci..

[73] Alharbi F.N., Abaker Z.M., Makawi S.Z.A. (2023). Phytochemical substances—Mediated synthesis of zinc oxide nanoparticles (ZnO NPS). Inorganics.

[74] Hosseinzadeh E., Foroumadi A., Firoozpour L. (2023). What is the role of phytochemical compounds as capping agents for the inhibition of aggregation in the green synthesis of metal oxide nanoparticles? A DFT molecular level response. Inorg. Chem. Commun..

[75] Sharma A.R., Sharma G., Nath S., Lee S.-S. (2024). Screening the phytochemicals in *Perilla* leaves and phytosynthesis of bioactive silver nanoparticles for potential antioxidant and wound-healing application. Green Process. Synth..

[76] Mahajan M., Kumar S., Gaur J., Kaushal S., Dalal J., Singh G., Misra M., Ahlawat D.S. (2025). Green synthesis of ZnO nanoparticles using *Justicia adhatoda* for photocatalytic degradation of malachite green and reduction of 4-nitrophenol. RSC Adv..

[77] Venkateasan A., Prabakaran R., Sujatha V. (2017). Phytoextract-mediated synthesis of zinc oxide nanoparticles using aqueous leaves extract of *Ipomoea pes-caprae (L).R.br* revealing its biological properties and photocatalytic activity. Nanotechnol. Environ. Eng..

[78] Oluwaniyi O.O., Oyewo B.T. (2024). Green Synthesis of Zinc Oxide nanoparticles via *Dennettia tripetala* extracts: Optimization, characterization, and biological activity evaluation. Nano Sel..

[79] Das R.K., Borthakur B.B., Bora U. (2010). Green synthesis of gold nanoparticles using ethanolic leaf extract of *Centella asiatica*. Mater. Lett..

[80] Pham X.N., Nguyen H.T., Pham N.T. (2020). Green synthesis and antibacterial activity of HAp@Ag nanocomposite using *Centella asiatica* (L.) Urban extract and eggshell. Int. J. Biomater..

[81] Adhavan R., Selvam K., Prakash P., Manimegalai P., Kirubakaran D., Shivakumar M.S. (2024). Bioefficacy of zinc oxide nanoparticle synthesis and their biological, environmental applications from *Eranthemum roseum*. Toxicol. Rep..

[82] Gnanasekaran R., Yuvaraj D., Reddy G.K., Shangar S.N., Vijayakumar V., Iyyappan J. (2024). Zinc oxide nanoparticles from leaf extract of *Eclipta prostrata*: Biosynthesis and characterization towards potential agent against film forming bacteria in metal working fluids. Environ. Chem. Ecotoxicol..

[83] Fernandes C.A., Jesudoss M.N., Nizam A., Krishna S.B.N., Lakshmaiah V.V. (2023). Biogenic synthesis of zinc oxide nanoparticles mediated by the extract of *Terminalia catappa* fruit pericarp and its multifaceted applications. ACS Omega.

[84] Bhattacharjee S. (2016). DLS and zeta potential—What they are and what they are not?. J. Control. Release.

[85] Bandeira M., Giovanela M., Roesch-Ely M., Devine D.M., da Silva Crespo J. (2020). Green synthesis of zinc oxide nanoparticles: A review of the synthesis methodology and mechanism of formation. Sustain. Chem. Pharm..

[86] Mongy Y., Shalaby T. (2024). Green synthesis of zinc oxide nanoparticles using *Rhus coriaria* extract and their anticancer activity against triple-negative breast cancer cells. Sci. Rep..

[87] Jan H., Shah M., Usman H., Khan M.A., Zia M., Hano C., Abbasi B.H. (2020). Biogenic synthesis and characterization of antimicrobial and antiparasitic zinc oxide (ZnO) nanoparticles using aqueous extracts of the Himalayan columbine (*Aquilegia pubiflora*). Front. Mater..

[88] Selvanathan V., Aminuzzaman M., Tan L.X., Win Y.F., Guan Cheah E.S., Heng M.H., Tey L.-H., Arullappan S., Algethami N., Alharthi S.S. (2022). Synthesis, characterization, and preliminary *in vitro* antibacterial evaluation of ZnO nanoparticles derived from soursop (*Annona muricata* L.) leaf extract as a green reducing agent. J. Mater. Res. Technol..

[89] Bin Ali M., Iftikhar T., Majeed H. (2024). Green synthesis of zinc oxide nanoparticles for the industrial biofortification of (*Pleurotus pulmonarius*) mushrooms. Heliyon.

[90] Iravani S. (2011). Green synthesis of metal nanoparticles using plants. Green Chem..

[91] Ahmed S., Ahmad M., Swami B.L., Ikram S. (2016). A review on plants extract mediated synthesis of silver nanoparticles for antimicrobial applications: A green expertise. J. Adv. Res..

[92] Lide D.R. (2005). CRC Handbook of Chemistry and Physics.

[93] Rice-Evans C., Miller N., Paganga G. (1997). Antioxidant properties of phenolic compounds. Trends Plant Sci..

[94] Shahidi F., Ambigaipalan P. (2015). Phenolics and polyphenolics in foods, beverages and spices: Antioxidant activity and health effects—A review. J. Funct. Foods.

[95] Shobha B., Lakshmeesha T.R., Ansari M.A., Almatroudi A., Alzohairy M.A., Basavaraju S., Alurappa R., Niranjana S.R., Chowdappa S. (2020). Mycosynthesis of ZnO nanoparticles using *Trichoderma* spp. isolated from rhizosphere soils and its synergistic antibacterial effect against *Xanthomonas oryzae* pv. *oryzae*. J. Fungi.

[96] Trzcińska-Wencel J., Wypij M., Terzyk A.P., Rai M., Golińska P. (2023). Biofabrication of novel silver and zinc oxide nanoparticles from *Fusarium solani* IOR 825 and their potential application in agriculture as biocontrol agents of phytopathogens, and seed germination and seedling growth promoters. Front. Chem..

[97] Siddiqi K.S., ur Rahman A., Tajuddin N., Husen A. (2018). Properties of zinc oxide nanoparticles and their activity against microbes. Nanoscale Res. Lett..

[98] Liang Y., Yang D., Cui J. (2017). A graphene oxide/silver nanoparticle composite as a novel agricultural antibacterial agent against *Xanthomonas oryzae* pv. *oryzae* for crop disease management. New J. Chem..

[99] Majumdar T.D., Singh M., Thapa M., Dutta M., Mukherjee A., Ghosh C.K. (2019). Size-dependent antibacterial activity of copper nanoparticles against *Xanthomonas oryzae* pv. *oryzae*—A synthetic and mechanistic approach. Colloids Interface Sci. Commun..

[100] Namburi K.R., Kora A.J., Chetukuri A., Kota V.S.M.K. (2021). Biogenic silver nanoparticles as an antibacterial agent against bacterial leaf blight causing rice phytopathogen *Xanthomonas oryzae* pv. *oryzae*. Bioprocess Biosyst. Eng..

[101] Tiwari V., Mishra N., Gadani K., Solanki P.S., Shah N.A., Tiwari M. (2018). Mechanism of anti-bacterial activity of zinc oxide nanoparticle against carbapenem-resistant *Acinetobacter baumannii*. Front. Microbiol..

[102] Babayevska N., Przysiecka Ł., Iatsunskyi I., Nowaczyk G., Jarek M., Janiszewska E., Jurga S. (2022). ZnO size and shape effect on antibacterial activity and cytotoxicity profile. Sci. Rep..

[103] Ahmed K.B.A., Anbazhagan V. (2017). Synthesis of copper sulfide nanoparticles and evaluation of in vitro antibacterial activity and in vivo therapeutic effect in bacteria-infected zebrafish. RSC Adv..

[104] Wei Y., Wang J., Wu S., Zhou R., Zhang K., Zhang Z., Liu J., Qin S., Shi J. (2022). Nanomaterial-based zinc ion interference therapy to combat bacterial infections. Front. Immunol..

[105] Li M., Zhu L., Lin D. (2011). Toxicity of ZnO nanoparticles to *Escherichia coli*: Mechanism and the influence of medium components. Environ. Sci. Technol..

[106] Rashid M.H.-O., Akter M.M., Uddin J., Islam S., Rahman M., Jahan K., Sarker M.M.R., Sadik G. (2023). Antioxidant, cytotoxic, antibacterial and thrombolytic activities of *Centella asiatica* L.: Possible role of phenolics and flavonoids. Clin. Phytosci..

[107] Liu Y., Zhu J., Liu Z., Zhi Y., Mei C., Wang H. (2025). Flavonoids as promising natural compounds for combating bacterial infections. Int. J. Mol. Sci..

[108] Kaushik M., Niranjan R., Thangam R., Madhan B., Pandiyarasan V., Ramachandran C., Oh D.-H., Venkatasubbu G.D. (2019). Investigations on the antimicrobial activity and wound healing potential of ZnO nanoparticles. Appl. Surf. Sci..

[109] Aydin Acar C., Gencer M.A., Pehlivanoglu S., Yesilot S., Donmez S. (2024). Green and eco-friendly biosynthesis of zinc oxide nanoparticles using *Calendula officinalis* flower extract: Wound healing potential and antioxidant activity. Int. Wound J..

[110] Selim M.I., Sonbol F.I., El-Banna T.E., Negm W.A., Elekhnawy E. (2024). Antibacterial and wound healing potential of biosynthesized zinc oxide nanoparticles against carbapenem-resistant *Acinetobacter baumannii*: An in vitro and in vivo study. Microb. Cell Fact..

[111] Tan Y., Hu A., Lu J., Lin Y., Li X., Yamaguchi T., Tabuchi M., Kawakami Z., Ikarashi Y., Kobayashi H. (2025). Protective effects of *Centella asiatica* against senescence and apoptosis in epidermal cells. Biology.

