# Supplementary material for: Plant-Based ZnO Nanoparticles for Green Nanobiocontrol of a Highly Virulent Bacterial Leaf Blight Pathogen: Mechanistic Insights and Biocompatibility Evaluation
Source: Nanomaterials (Basel). 2025 Jun 30;15(13):1011. doi: 10.3390/nano15131011 (PMC12251038; doi:10.3390/nano15131011)
Supplement: Supplementary file 1 [file nanomaterials-15-01011-s001.zip › nanomaterials-3650068-supplementary.pdf]

nanomaterials

Supplementary Materials

Plant-Based ZnO Nanoparticles for Green Nanobiocontrol of a Highly Virulent Bacterial Leaf Blight Pathogen: Mechanistic Insights and Biocompatibility Evaluation

Preeda Chanthapong [1], Duangkamol Maensiri [2,*], Paweena Rangsrisak [1], Thanee Jaiyan [1], Kanchit Rahaeng [1], Atcha Oraintara [3], Kunthaya Ratchaphonsaenwong [4], Jirawat Sanitchon [5], Piyada Theerakulpisut [1] and Wuttipong Mahakham [1,*]

[1] Department of Biology, Faculty of Science, Khon Kaen University, Khon Kaen 40002, Thailand; jirapat@kku.ac.th (P.C.); rpaweena@kkumail.com (P.R.); thaneeja@kkumail.com (T.J.); kanchit.ra@kkumail.com (K.R.); piythe@kku.ac.th (P.T.)
[2] School of Biology, Institute of Science, Suranaree University of Technology, Nakhon Ratchasima 30000, Thailand
[3] Department of Microbiology, Faculty of Science, Khon Kaen University, Khon Kaen 40002, Thailand; atcha@kku.ac.th
[4] Research and Academic Services Division, Faculty of Science, Khon Kaen University, Khon Kaen 40002, Thailand; phikra@kku.ac.th
[5] Department of Agronomy, Faculty of Agriculture, Khon Kaen University, Khon Kaen 40002, Thailand; jirawat@kku.ac.th
* Correspondence: duangkamol@sut.ac.th (D.M.); mwuthi@kku.ac.th (W.M.)

Academic Editor(s): Jose L. Arias

Received: 2 May 2025
Revised: 19 June 2025
Accepted: 24 June 2025
Published: 30 June 2025

Citation: Chanthapong, P.; Maensiri, D.; Rangsrisak, P.; Jaiyan, T.; Rahaeng, K.; Oraintara, A.; Ratchaphonsaenwong, K.; Sanitchon, J.; Theerakulpisut, P.; Mahakham, W. Plant-Based ZnO Nanoparticles for Green Nanobiocontrol of a Highly Virulent Bacterial Leaf Blight Pathogen: Mechanistic Insights and Biocompatibility Evaluation. *Nanomaterials* **2025**, *15*, x. https://doi.org/10.3390/xxxxx

Copyright: © 2025 by the authors. Submitted for possible open access publication under the terms and conditions of the Creative Commons Attribution (CC BY) license (https://creativecommons.org/licenses/by/4.0/).

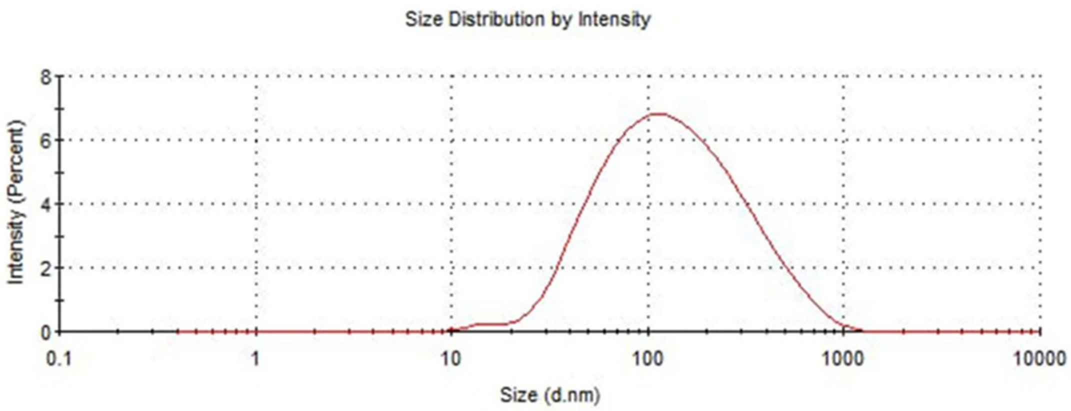

Figure S1. *Dynamic light scattering (DLS) size distribution of CA-ZnO NPs dispersed in deionized water.* The intensity-based size distribution reveals a bimodal profile with a major peak at 176.7 nm and a minor peak at 14.8 nm. The Z-average hydrodynamic diameter was 98.94 nm, and the polydispersity index (PDI) was 0.386, indicating moderate size heterogeneity.

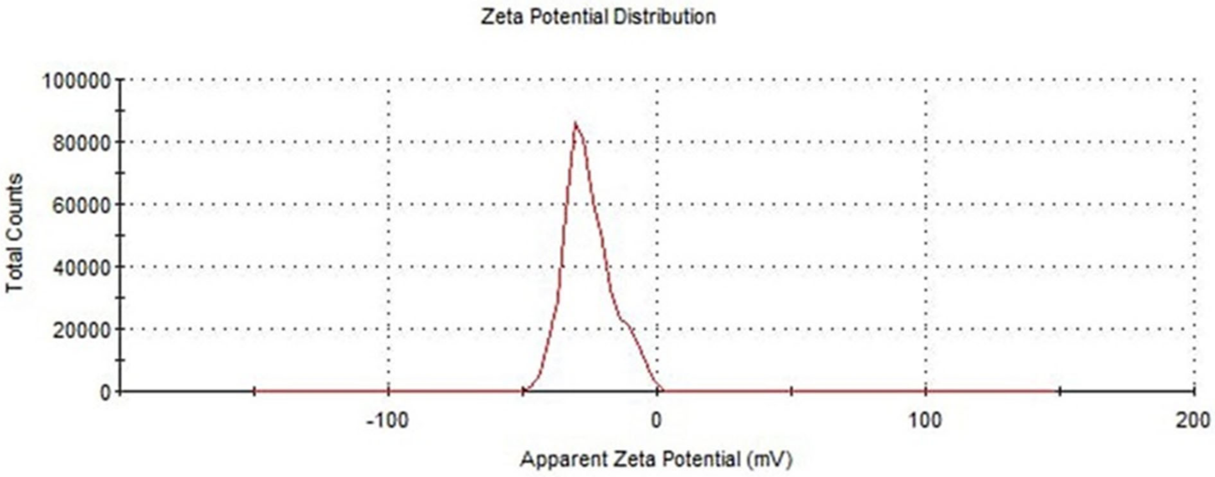

Figure S2. *Zeta potential measurement of CA-ZnO NPs in deionized water.* The mean zeta potential was −25.5 mV with a standard deviation of ±8.87 mV, suggesting moderate colloidal stability due to electrostatic repulsion between negatively charged particles.
